# Topic Application of the Probiotic *Streptococcus dentisani* Improves Clinical and Microbiological Parameters Associated With Oral Health

**DOI:** 10.3389/fcimb.2020.00465

**Published:** 2020-08-31

**Authors:** María D. Ferrer, Aranzazu López-López, Teodora Nicolescu, Salvadora Perez-Vilaplana, Alba Boix-Amorós, Majda Dzidic, Sandra Garcia, Alejandro Artacho, Carmen Llena, Alex Mira

**Affiliations:** ^1^Foundation for the Promotion of Health and Biomedical Research of Valencia Region (FISABIO), Valencia, Spain; ^2^Clínica Odontológica, Fundació Lluís Alcanyis, Universitat de València, Valencia, Spain

**Keywords:** probiotic, bacteriocin, dental plaque, salivary flow, dental caries

## Abstract

*Streptococcus dentisani* 7746, isolated from dental plaque of caries-free individuals, has been shown to have several beneficial effects *in vitro* which could contribute to promote oral health, including an antimicrobial activity against oral pathogens by the production of bacteriocins and a pH buffering capacity through ammonia production. Previous work has shown that *S. dentisani* was able to colonize the oral cavity for 2–4 weeks after application. The aim of the present work was to evaluate its clinical efficacy by a randomized, double-blind, placebo-controlled parallel group study. Fifty nine volunteers were enrolled in the study and randomly assigned to a treatment or placebo group. The treatment consisted of a bucco-adhesive gel application (2.5 10^9^ cfu/dose) with a dental splint for 5 min every 48 h, for a period of 1 month (i.e., 14 doses). Dental plaque and saliva samples were collected at baseline, 15 and 30 days after first application, and 15 days after the end of treatment. At baseline, there was a significant correlation between *S. dentisani* levels and frequency of toothbrushing. Salivary flow, a major factor influencing oral health, was significantly higher in the probiotic group at day 15 compared with the placebo (4.4 and 3.4 ml/5 min, respectively). In the probiotic group, there was a decrease in the amount of dental plaque and in gingival inflammation, but no differences were observed in the placebo group. The probiotic group showed a significant increase in the levels of salivary ammonia and calcium. Finally, Illumina sequencing of plaque samples showed a beneficial shift in bacterial composition at day 30 relative to baseline, with a reduction of several cariogenic organisms and the key players in plaque formation, probably as a result of bacteriocins production. Only 58% of the participants in the probiotic group showed increased plaque levels of *S. dentisani* at day 30 and 71% by day 45, indicating that the benefits of *S. dentisani* application could be augmented by improving colonization efficiency. In conclusion, the application of *S. dentisani* 7746 improved several clinical and microbiological parameters associated with oral health, supporting its use as a probiotic to prevent tooth decay.

## Introduction

Oral diseases, including dental caries (tooth decay), periodontitis (gum disease), and halitosis (bad breadth) are among the most prevalent diseases worldwide (Petersen, 2008). Although all these conditions are caused by microorganisms, current data indicate that they are not caused by exogenously acquired organisms, as healthy individuals already contain low levels of the microorganisms identified as causing agents and oral diseases therefore do not develop as a consequence of being infected by those microbes (Bradshaw and Lynch, 2013; Camelo-Castillo et al., 2015; Simón-Soro and Mira, 2015). Instead, they appear to be the consequence of external disease drivers that break the host-microbiota homeostasis (Marsh, 2003). These triggering factors range from a sugar-rich diet in the case of dental caries to bad oral hygiene and dental plaque accumulation in the case of periodontal diseases (Rosier et al., 2018). For instance, dietary sugars can be fermented by saccharolytic oral bacteria, producing organic acids that lower the pH and break the ecosystem balance, as acidogenic and aciduric organisms increase in proportion at the expense of a reduced diversity (Bradshaw et al., 2002). As a consequence of this state of dysbiosis, the pH may drop below a threshold where enamel tissue demineralizes, giving rise to a caries lesion (Takahashi and Nyvad, 2011). Although the oral cavity has several immunological and physico-chemical mechanisms that confer some resilience to disease (Rosier et al., 2018), these vary widely among individuals and external intervention is needed to prevent the development of oral diseases. However, the coexistence with pathogenic species in healthy individuals and the polymicrobial nature of dental caries suggest that immunization strategies such as a caries vaccine directed to a single species are unlikely to be effective (Simón-Soro and Mira, 2015; Mira, 2018). Similarly, indiscriminate antimicrobial or antiseptic strategies eliminate the beneficial action of oral microorganisms, leading to unexpected outcomes like an increase in blood pressure as a consequence of a reduction in nitric oxide availability (Bondonno et al., 2015). Eliminating oral communities may also disrupt the microorganism's ability to inhibit pathogenic species, favoring the colonization of opportunistic pathogens such as *Candida* species (Bertolini et al., 2019). Thus, recent views in preventive dentistry suggest shifting from antimicrobial strategies toward the development of products that can restore the balance in the oral ecosystem (Marsh, 2018; Mira, 2018), and these include the use of prebiotics and probiotics (Koduganti et al., 2011).

Initially, the success of probiotics isolated from dairy products or from the gut of humans and animals to improve physiological functions in the gastrointestinal tract or the immune system, led to the application of the same or similar species to treat oral diseases (Allaker and Stephen, 2017). However, different clinical studies have shown that oral colonization is extremely limited in these non-oral probiotics (Ravn et al., 2012; Taipale et al., 2012; Zaura and Twetman, 2019). In addition, many classical probiotic species, especially Lactobacilli and Bifidobacteria, are highly acidogenic and therefore related to dental caries initiation or progression (Badet and Thebaud, 2008; Mantzourani et al., 2009), and *in vitro* studies have shown that they can increase the acidogenicity of oral biofilms (Pham et al., 2009). For these reasons, the use of health-associated oral bacteria with potentially beneficial features has been suggested to prevent and treat oral diseases (Nascimento et al., 2013; López-López et al., 2017; Marsh, 2017; Mira, 2018). Some of those beneficial characteristics have consequences for systemic health, like the metabolic conversion of nitrate into nitrite and nitric oxide, which influences cardiovascular function (Doel et al., 2005). In relation to the promotion of oral health, some oral bacteria have been found to buffer extracellular pH by ammonia production, through the arginolytic, urease, or denitrification pathways (Liu et al., 2012; Reyes et al., 2014; Rosier et al., 2018) and would therefore be especially useful to prevent tooth decay. In other occasions anti-inflammatory properties have been reported (Corrêa et al., 2019; Esteban-Fernández et al., 2019) which could thus be relevant for gingivitis and periodontitis. Different bacterial species have also been found to have antimicrobial properties, with antagonistic effects against oral pathogens. These include many streptococcal species such as *S. salivarius, S. dentisani, Streptococcus* A12, and *S. oligofermentans*, which inhibit the growth of different oral pathogenic strains by the production of hydrogen peroxide, proteases, or bacteriocins (Burton et al., 2006; Bao et al., 2015; Huang et al., 2016; Llena et al., 2019). Although the potential benefits of these oral organisms are promising and they appear to be associated to individuals with good oral health (Belda-Ferre et al., 2012; Liu et al., 2012), there is still limited clinical evidence of their colonization efficacy, *in vivo* benefits for human health, side effects and consequences for oral microbial ecology (Allaker and Stephen, 2017; Zaura and Twetman, 2019). Thus, clinical studies aiming at testing oral probiotics' efficacy to promote oral health are vital for evaluating their use.

*Streptococcus dentisani* was first isolated in 2011 from caries-free individuals where a metagenomic analysis of dental plaque samples had shown an over-representation of genes coding for antimicrobial peptides (Belda-Ferre et al., 2012). In fact, at least 11 bacteriocins have been described in this organism based on bioinformatic analysis (Conrads et al., 2019) and has been shown to inhibit different oral pathogens ranging from *Fusobacterium nucleatum* to *Porphyromonas gingivalis* or *Streptococcus mutans* (López-López et al., 2017; Esteban-Fernández et al., 2019; Llena et al., 2019). Genomic and physiological analysis determined that this bacterium represented a new species (Benítez-Páez et al., 2014) although its potential assignment as a subspecies of *S. oralis* is under debate (Jensen et al., 2016; Velsko et al., 2019). Nevertheless, over a dozen isolates have now been sequenced and their phylogenomic analysis shows that they form a clearly distinct cluster (Jensen et al., 2016), being the presence of the arginolytic pathway a landmark of this taxon that further differentiates it from related *S. oralis* and *S. tigurinus* clusters which do not have this pathway. The current official assignment for this cluster is *S. oralis* subspecies *dentisani* and given that *S. dentisani* is considered a synonym, we will use this term throughout the manuscript. *In vitro* studies show that *S. dentisani* is able to inhibit and displace pre-existing cultures of oral pathogens on human gingival cells (Esteban-Fernández et al., 2018, 2019). Recently, a pilot clinical study showed that *S. dentisani* strain 7746 was able to colonize human dental plaque for at least 2 weeks after topic application with a dental ferule (Ferrer et al., 2020). In addition, salivary pH and lactate production improved after 1-week treatment, and a reduction in the counts of the cariogenic organism *S. mutans* was also observed, but the small sample size in this pilot test (only 12 individuals were studied) prevented a full evaluation of this strain as a probiotic. In the current manuscript, we aim to evaluate the clinical efficacy of *S. dentisani* 7746 by a randomized, double-blind, placebo-controlled parallel group study. Both saliva and plaque samples were collected at different time points for measuring different physico-chemical (e.g., pH, lactate, calcium, or ammonia, among others) and microbiological features, including a full bacterial composition analysis by high-throughput 16S rRNA gene sequencing. By these data, together with the analysis of clinical parameters that are associated with oral health, such as plaque accumulation, gingival inflammation, oral hygiene, or salivary flow, we intend to provide the first complete evaluation of the *in vivo* efficacy and safety of this organism.

## Materials and Methods

### Study Design, Recruitment, and Treatment

To evaluate the protective effect on dental caries of *Streptococcus dentisani* CECT 7746, a randomized, double-blind, placebo-controlled parallel group clinical trial was designed (ClinicalTrials.gov Identifier: NCT03468842). The present study (project code ABB-dentisani 2015) was approved on 2015/12/30th by the Institutional Ethical Committee DGSP-CSISP. Compliance of the protocol and surveillance of the study was performed by the external CRO Effice (Madrid, Spain).

Sample size was calculated by a power estimation based on the pH variable, considering a pH variation in the population of 0.5 and an expected difference between the groups of 0.5, a confidence level of 95% and a power of 90%, which suggested a sample size of 22 individuals per group. We expected a high dropout rate (>20%) due to the length of the study and the multiple visits, we aimed at a final sample size of 55–60 individuals. One hundred eighty-four patients were recruited from which 59 subjects (41 females and 18 males) aged 18–65 years fulfilled inclusion/exclusion criteria and were enrolled in the study. Inclusion criteria included three to ten carious active lesions (including white spot lesions and non-cavitated lesions on enamel surface), with ICDAS score of 2 to 5. Exclusion criteria included presence of <20 teeth, periodontal diseases, oral antiseptics use or oral probiotics or antibiotics consumption during the 30 days before the start of the study, chronic diseases, or chronic treatment that can reduce salivary flow (e.g., psychotropic drugs or antidepressants) pregnant or lactating women and allergy to any of the product composition ingredients. Individuals with a salivary pH >7 were excluded in order to select for patients with a higher caries risk, which would be the ones benefiting the most from an anti-caries treatment. The number of tooth brushings per day before the study was registered for all participants (0, 1, 2, or 3).

Participants, after signing the informed consent form, were randomly assigned by a computer-generated list to one of the two treatment groups (29 patients in the placebo group and 30 patients in the probiotic group). Soft personalized splints necessary for product application were manufactured based on alginate impressions from both jaw arches of all participants. These splints, washable and reusable during the entire study, were provided to participants together with a toothpaste containing 1,450 ppm fluoride (supplied by AB-Biotics SA) and a toothbrush. To ensure homogenous dental hygiene among participants, volunteers were instructed how to brush their teeth twice a day (morning and night) and the amount of dentifrice to use. Participants were asked to refrain from tooth brushing the morning of sampling days (day 0, day 15, day 30, and day 45) to increase plaque accumulation for sampling and to homogenize plaque formation time. No dietary recommendations were provided.

**Figure 1:**
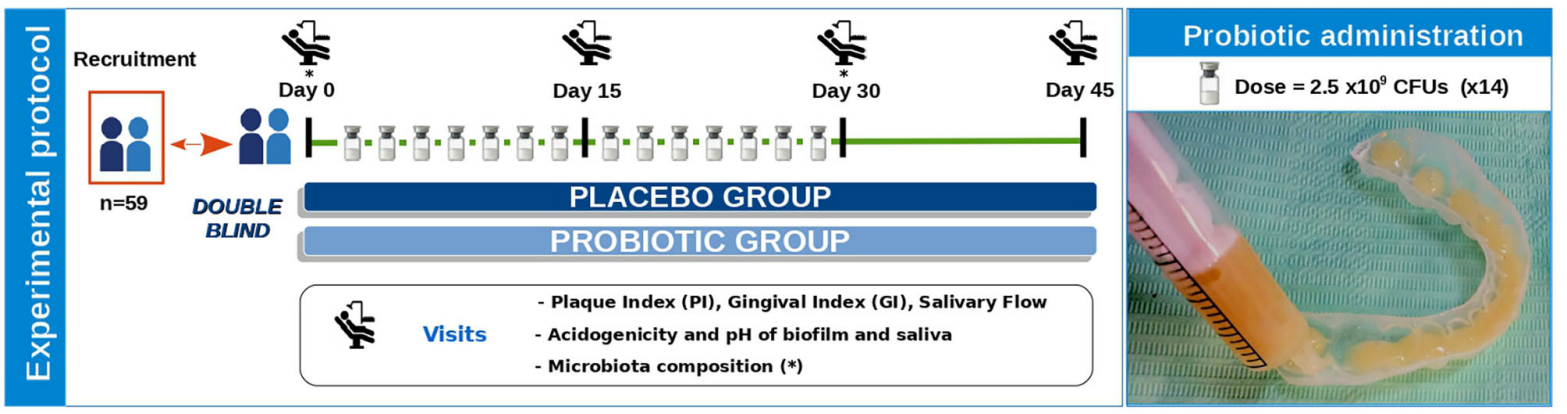
Diagram of clinical study experimental protocol. In this double-blind study, participants were randomly assigned to one of the two treatment groups (placebo or probiotic). On the first visit (Day 0), treatment was applied by the dentist and participants were explained how to perform the 14 successive applications at home every 48 h during a month. At each of the visits (Day 0, Day 15, Day 30, and Day 45), several clinical parameters were measured (plaque and gingival index, and salivary flow), and plaque and saliva samples were collected to determine the acidogenicity and buffer capacity of biofilm and saliva. All samples at day 0 were collected before any administration of the probiotic or placebo and therefore *S. dentisani* levels at day 0 correspond to the endogenous levels of this organism in the participants. The image on the right shows bucco-adhesive product administration. Lyophilized *S. dentisani* cells (with a total dose of 2.5 × 10^9^ CFUs) mixed with thickener and adherence excipients were resuspended with 6 mL of water, and resultant gel was transferred to the individualized mouth splint using a syringe. The placebo group product had the same excipients but no probiotic.

At day 0, treatment was applied by the dentist and the participants were explained how to perform the successive applications at home. Volunteers received the probiotic/placebo vials for home applications, which were performed every 48 h for a month (14 applications in total). At the follow-up visits (days 15, 30, and 45), plaque and saliva sampling procedures were followed as in the baseline visit (see below). Compliance was confirmed by checking the empty vials in visit 30 when patients returned the used material. Figure 1 (left panel) shows a scheme of the study design and visits. Compliance with the protocol, follow-up of participants, data notebook, and congruence of the source documents with clinical and laboratory data were performed and supervised by the external CRO Effice Ltd. (Madrid, Spain). Effice Ltd. was also responsible for configuring the database and breaking the blindness after all clinical and laboratory data were registered.

### Experimental Product

Lyophilized *S. dentisani* cells of strain CECT 7746 were provided in sealed glass vials mixed with adherent and thickener excipients. Probiotic and placebo composition are shown in Table 1. The dry product was resuspended with 6 ml of sterile water, and after 30 s of shaking, the resulting bucco-adhesive gel was transferred to the individualized tailored mouth splints (3 ml on each) using a sterile syringe, and then applied in contact with the surface of all teeth for 5 min (Figure 1, right panel). Participants were explicitly asked to refrain from drinking, eating, or toothbrushing for at least half an hour after the product's application.

**Table 1:** Experimental product.

	**Probiotic**	**Placebo**
General content	Probiotic *Streptococcus dentisani* CECT 7746 + Excipients	Excipients without probiotic
Vial composition	-2.5 × 10^9^ CFUs/vial - 2% (p/v) Guar gum - 6% (p/v) hydroxyethylcellulose	-2% (p/v) Guar gum - 6% (p/v) hydroxyethylcellulose
Probiotic dose	2.5 × 10^9^ CFUs/vial Administration every 48 h, equivalent to 10^10^ CFUs/week	–
Route of administration	Topic application in the mouth	Topic application in the mouth
Galenic form	Buccoadhesive gel	Buccoadhesive gel

*Probiotic and placebo composition*.

### Collection of Oral Samples

Visits took place in between 4 and 7 p.m. In order to accumulate a mature dental biofilm of 16–20 h, volunteers refrained from brushing their teeth since the night before. Plaque samples were collected separately from quadrants 1 to 3 and 2 to 4 in 100 μl of PBS and stored on ice. Supragingival dental plaque was collected from lingual and vestibular surfaces of all teeth with sterile spoon excavators, following Simón-Soro et al. (2013). For saliva samples, in every sampling event, three different non-stimulated, drooling saliva samples were collected in 50 ml sterile Falcon tubes and kept on ice: basal saliva (intact), saliva after tooth brushing with water, and saliva collected 10 min after a minute sugar rinse with 10 ml of 10% sucrose solution. Salivary samples taken for measuring pH and lactate were processed and measured immediately. The surplus of saliva samples was kept on ice during the clinical session, and all samples were transported to the lab and kept at −20°C until processing.

### Clinical Parameters

Dental examinations were performed by an experienced, ICDAS-certified dentist. An odontogram and DMFT index were registered for all recruited individuals at baseline. In every visit, and prior to plaque sample collection, three parameters were measured: plaque index (PI), gingival index (GI), and salivary flow. The plaque index was determined in the surface of six different teeth by the Silnes and Löe methodology (Sillness and Löe, 1964). The gingival index was determined in the same six positions by the Löe and Silnes methodology (Löe and Silness, 1963). For each index and volunteer, means of the values obtained in the six teeth were calculated. To estimate the salivary flow, volunteers deposited the drooling unstimulated saliva in a graduated tube during 5 min (Navazesh, 1993).

### Lactic Acid and pH Measurements

To measure total lactic acid and pH of saliva and plaque samples, reflectometry with the RQflex20 Reflectoquant System (Merk) was used, according to manufacturer's instructions and following Helmke et al. (2009) and Ferrer et al. (2020). The equipment was calibrated by the use of standards with known concentrations.

A plaque sugar challenge was performed by adding 20 μl of sucrose 20% in PBS to an aliquot of 20 μl directly taken form the plaque suspension. After an incubation period of 10 min at 37°C, *ex vivo* plaque pH and lactic acid measurements were obtained by reflectometry as explained before.

### Electrolytes Quantification

The majority ions in the basal saliva samples taken during the visit at beginning of the treatment (V0) and at the end of the treatment (V30) were determined by ion chromatography in Metrohm Com-pact IC Plus 882 equipment. Each determination was made in duplicate. Six anions (Fluoride, chlorine, nitrite, nitrate, phosphate, and sulfate) and five cations (sodium, ammonium, potassium, calcium, and magnesium) were quantified.

### Nucleic Acids Extraction

An aliquot of 250 μl of each saliva sample was thawed and centrifuged at 13.000 rpm for 10 min, and the pellet was resuspended in 100 μl of PBS. Dental plaque samples were mixed by vigorous shaking. Then both kind of samples were immersed in an ultrasounds bath (Selecta) during 45 seg. Before starting DNA extraction with the MagnaPure LC JE379 instrument and the MagnaPure LC DNA Isolation Kit (Roche) following the manufacturer's instructions, an additional enzymatic lysis step was performed as published before (Dzidic et al., 2018). DNA extracted was quantified in duplicate with the Quant-iT PicoGreen dsDNA Assay Kit (Invitrogen).

### Quantification of *S. dentisani* in Dental Plaque and Saliva Samples

*Streptococcus dentisani* in saliva and dental plaque samples was quantified by real-time PCR at each study visit. *S. dentisani* primers targeted the genes for the carbamate kinase (*arcC*), as detailed in Ferrer et al. (2020). Real-time PCR was performed in a LightCycler 480 System with the LightCycler 480 SYBR Green I Master Mix (Roche).

The absolute amounts of *S. dentisani* were calculated by comparison with the Cq values obtained from a standard curve, using serial ten-fold dilutions of DNA extracted from 2 × 10^6^ CFUs/ml of *S. dentisani* (counted by serial dilutions in agar plates). Cq values obtained from plaque samples were expressed in absolute numbers per two quadrants or normalized against the total ng of DNA of plaque, whereas in saliva samples *S. dentisani* cells were normalized by ml of saliva.

### 16S rDNA Gene Amplification, Sequencing, and Data Analysis

Illumina amplicon libraries were performed following the 16S rDNA gene Metagenomic Sequencing Library Preparation Illumina protocol (Part #15044223 Rev. A). The universal primer targets the 16S rDNA gene V3 and V4 regions, resulting in a single amplicon of 460 bp (Dzidic et al., 2018). Overhand adapter sequences were used together with the primer pair sequences for compatibility with Illumina index and sequencing adapters. Amplicons were sequenced on a MiSeq equipment according to manufacturer's instructions (Illumina) using the 2 × 300 bp paired-end protocol. Sequences were uploaded to the SRA public repository with accession number PRJNA629283.

Reads were analyzed as previously described (Carda-Diéguez et al., 2019) using the Dada2 pipeline (Callahan et al., 2016). Briefly, we filtered, end-trimmed, denoise, and merge paired reads before adapters and primers were filtered out. Singletons and PCR chimeras were removed. The remaining reads were assigned to a taxon using the SILVA non-redundant database (Quast et al., 2013). For taxonomic analysis, we obtained an amplicon sequence variant (ASV) table following the dada2 pipeline, assigning a taxonomy to each variant by comparing them against SILVA database with a naive Bayesian classifier implemented in R (R Core Team, 2013) to assign up to genus level and 97% blast matching for species level (ASV).

#### Statistical Analysis

The obtained values were plotted and subjected to a multiple *t*-test or One-way ANOVA test using GraphPad Prism version 6.0., depending on data normality, to reveal significant differences between sampling events, both intergroup and intragroup. The threshold for significance was set at *p-valor* ≤ 0.05. For taxonomic analysis, rarefaction curves, heatmaps, canonical correlation analysis (CCA), and Wilcoxon rank sum statistical tests were performed with R, using the packages Vegan (R Core Team, 2013) and ade4 (Dray and Dufour, 2007). When multiple comparisons were performed, *p*-values were corrected by the Bonferroni correction.

## Results

### Volunteers Dropout, Safety, and Tolerability

Of the 59 patients enrolled in the study, 52 completed the first visit during treatment (V15) there being 26 volunteers in each group. The dropout rate at the end of the study (V45) was 15.25% completing a total of 50 volunteers (25 in the placebo group and 25 in the probiotic group). At baseline, 20% of participants reported one toothbrushing per day, 48% reported two and 32% reported three times per day. Caries severity at baseline did not differ between the two groups, with mean DMFT values of 7.36 (*SD* 4.46) and 8.31 (*SD* 4–41) for probiotic and placebo groups, respectively. There were no serious adverse events. Mild events considered as having a potential relationship with the treatment included dyspepsia and abdominal pain and no significant difference in their frequency was found between the two groups.

### Clinical Parameters Associated With Oral Health

#### Plaque Index

**Figure 2:**
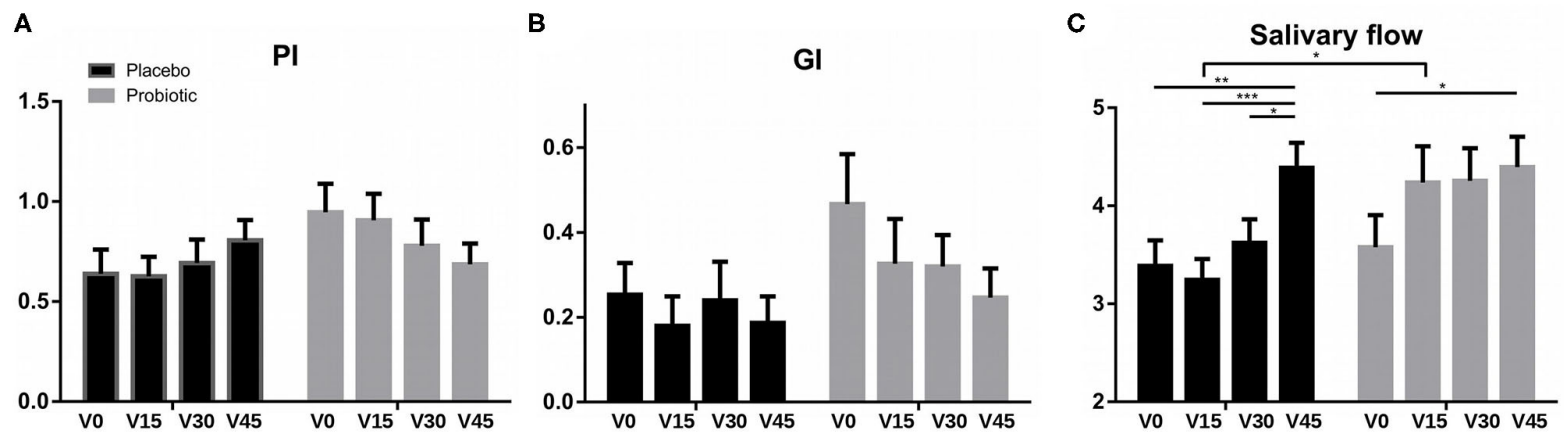
Clinical parameters. Data show average values of **(A)** plaque index (PI), **(B)** gingival index (GI), and **(C)** salivary flow in each group (placebo in black and probiotic group in gray) measured at the different visits. Bars, from left to right, represent mean values (SEM) at: V0, baseline visit; V15, 15 days of treatment; V30, end of treatment; and V45, 15 days after the end of treatment. *P < 0.05, **P < 0.01, and ***P < 0.001.

The average score of the plaque index (PI) was <1 in both groups during all the study (Figure 2A). Although the levels of plaque accumulation were therefore moderate, a progressive decrease in PI was observed between each visit throughout the study in the probiotic group, with a final score decrease of 0.26 points (V0 vs. V45). In contrast, in the placebo group, PI average values increased compared to baseline (V0), both at the end of treatment visit (V30), and at the end of the study (V45), and the increment value of PI between baseline and the end of the study (ΔPI V0–V45) was significantly higher compared to the probiotic group (Supplementary Figure 1A).

#### Gingival Index

The Figure 2B shows the mean GI values at each visit for the placebo and probiotic groups. Although both groups improved throughout the study, the decrease in GI was more pronounced in the probiotic group than in the placebo at each of the visits, but the difference was not statistically significant (Figure 2B and Supplementary Figure 1B).

#### Salivary Flow

The average values of unstimulated saliva produced during 5 min for the two treatment groups in each visit are represented in Figure 2C. In both groups, salivary flow increased significantly at the end of the study (V45) compared with baseline values (V0) (*p* ≤ 0.05). However, in the probiotic group this increase was already observed from the first visit (V15), progressively growing throughout the study (V0–V15: *p* = 0.06; V0–V30: *p* = 0.05). In the placebo group, an increase in salivary flow was only observed at the end of the study (V45). Regarding the differences between groups, the salivary flow of the probiotic group was significantly higher than the placebo after 15 days of treatment (V15), reaching an average flow of 4.24 ml compared to 3.24 ml in the placebo group (Figure 2C and Supplementary Figure 1C).

### *S. dentisani* Treatment Provides a Healthy Shift in Oral Microbiota Composition

The bacterial composition of supragingival dental plaque was studied before (V0) and at the end of treatment (V30) in probiotic and placebo groups. The relative abundance of the main bacterial genera was similar between the two study groups (Supplementary Figure 2A), indicating that the administration of *S. dentisani* did not trigger a dramatic ecological shift in the dental plaque microbiota. However, some interesting changes were observed, including a significant increase in *Streptococcus* in the probiotic group at the end of the treatment compared to the placebo group and to baseline values (*p* =0.021, Wilcox test) and a trend for a decrease in *Prevotella* (*p* = 0.068) and *Veillonella* (*p* = 0.088; Supplementary Figures 3A–C). Levels of each bacterial taxa in the two study groups at baseline and end-of-treatment timepoints, as well as their corresponding *p*-values are shown in Supplementary Table 1.

In terms of the composition at the genus level, a Canonical Correspondence Analysis (CCA) was used to identify the degree of similarity of the overall bacterial community composition between plaque samples (Supplementary Figure 2B), showing a large degree of overlap between placebo and probiotic groups at the end of the treatment (V30). However, two significant changes were observed for specific bacterial species in the probiotic group at the end of the treatment with respect to the other groups together (probiotic V0, placebo V0, and placebo V30): (i) the average values of *S. dentisani* were significantly higher in the patients after probiotic treatment (*p* = 0.006), being 3.8 % more abundant than in the other groups (Supplementary Figure 3D), and (ii) the average values of *Veillonella dispar*/*parvula* were 2,7% lower in the probiotic after treatment (*p* = 0.003; Supplementary Figure 3E).

**Figure 3:**
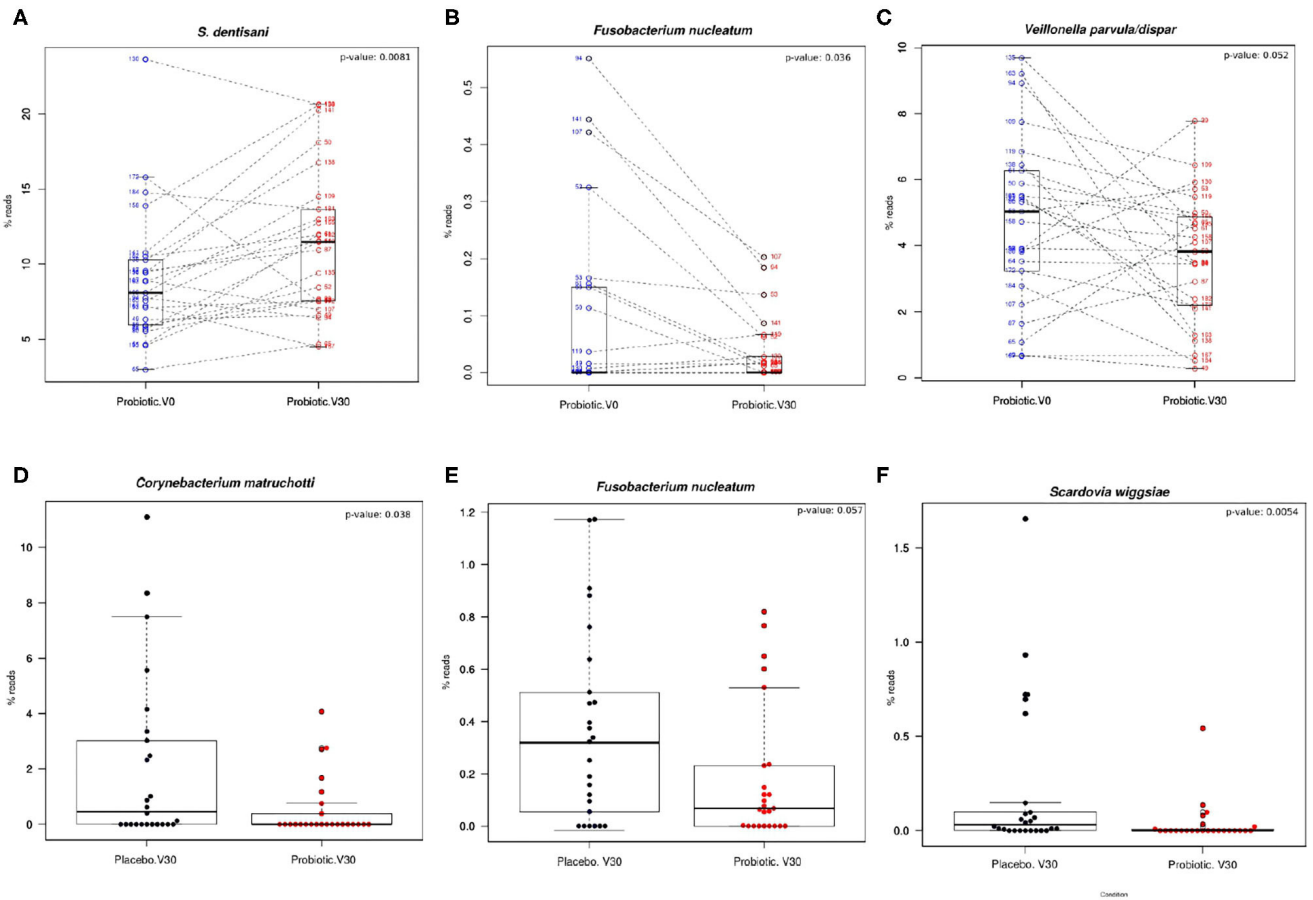
Shifts in the relative abundance in the most abundant bacteria in dental plaque. **(A–C)** Boxplots show levels of different bacterial species in the probiotic group before and after treatment (probiotic V0 vs. V30). Lines join the samples before and after the treatment period (paired samples). **(D–F)** Levels of different bacterial species in placebo and probiotic groups at the end of treatment (placebo V30 vs. probiotic V30). The relative abundance values of *Fusobacterium nucleatum* shown in **(B)** correspond to ASV 0216 and **(E)** to the reads of ASV 0090, respectively, both 99% identical to *F. nucleatum* type strain. The results of Wilcox paired test are also shown.

Within the probiotic group, a significant increase of *S. dentisani* (*p* = 0.008) was observed at the end of the treatment (Figure 3A), confirming colonization in dental plaque. In addition, a significant decrease in *Fusobacterium nucleatum* was observed (*p* = 0.032) after treatment with *S. dentisani* (Figure 3B). A trend was also observed in the decrease of *Veillonella dispar/párvula* (*p* = 0.052) (Figure 3C) suggesting a decrease in organic acids, given that these bacteria use lactate as carbon source.

Significant differences between probiotic and placebo groups at the end of the treatment were observed. Bacteria at higher levels in the placebo group compared to the probiotic, in order of significance, included *Scardovia wiggsiae* (Figure 3F), *Dialister invisus, Actinomyces* sp., *Capnocytophaga leadbetteri, Corynebacterium matruchotti* (Figure 3D) and *Prevotella denticola*; followed by *Fusobacterium nucleatum* (*p* = 0.057; Figure 3E) and *Atopobium parvulum* (*p* = 0.058). In contrast, the species at significantly higher levels in the probiotic group at the end of treatment compared to placebo comprised *Gemella morbillorum, Porphyromonas pasteri*, and *Kingella* sp.

Taken together, these results showed that treatment with *S. dentisani* probiotic results in a microbial shift in composition which is consistent with a healthier microbiota, as there is a decrease of microorganisms associated with dental caries such as *Scardovia wiggsiae* (Tanner et al., 2011), *Actinomyces* sp., *Veillonella* sp. (Belda-Ferre et al., 2012; Simón-Soro et al., 2014), and *Atopobium parvulum* (Obata et al., 2014); a decrease in bacteria associated with periodontal diseases and halitosis such as *Dialister* (Silva-Boghossian et al., 2013), *Prevotella* (Camelo-Castillo et al., 2015), and *Fusobacterium nucleatum* (Wang et al., 2019); and an increase in some bacteria associated with oral health such as *Gemella* (Luo et al., 2012) and *Kingella* (Belda-Ferre et al., 2012).

It is also interesting that the two bacterial species which are considered instrumental for dental plaque formation (*F. nucleatum*, Kolenbrander et al., 2006) and oral biofilm architecture (*C. matruchotii*, Ferrer and Mira, 2016) were found to significantly decrease at the end of *S. dentisani* probiotic treatment (Figures 3D,E), suggesting their possible involvement in lower plaque build-up. In congruence with the clinical measures of plaque index, plaque samples within the probiotic group showed a significant decrease (total bacteria/ng DNA; *p* = 0.002) in the amount of total bacteria (measured by qPCR on the 16S rRNA gene) normalized by ng of plaque DNA (as measured by fluorimetry) between baseline visit (V0) and the end of treatment. The data therefore suggest that the shift in bacterial composition hampered dental plaque accumulation.

### Calcium and Ammonium Salivary Levels Increase After Probiotic Application

In order to shed light on the possible cause of increased salivary flow observed during the probiotic treatment, 11 major electrolytes present in saliva were quantified before (V0) and after treatment (V30), to test whether a difference in osmotic pressure as a consequence of changes in electrolytes concentration could be favoring higher saliva production. However, no significant differences were observed between groups or between visits within each group in the cations and anions of the measured electrolytes (Supplementary Figure 4).

**Figure 4:**
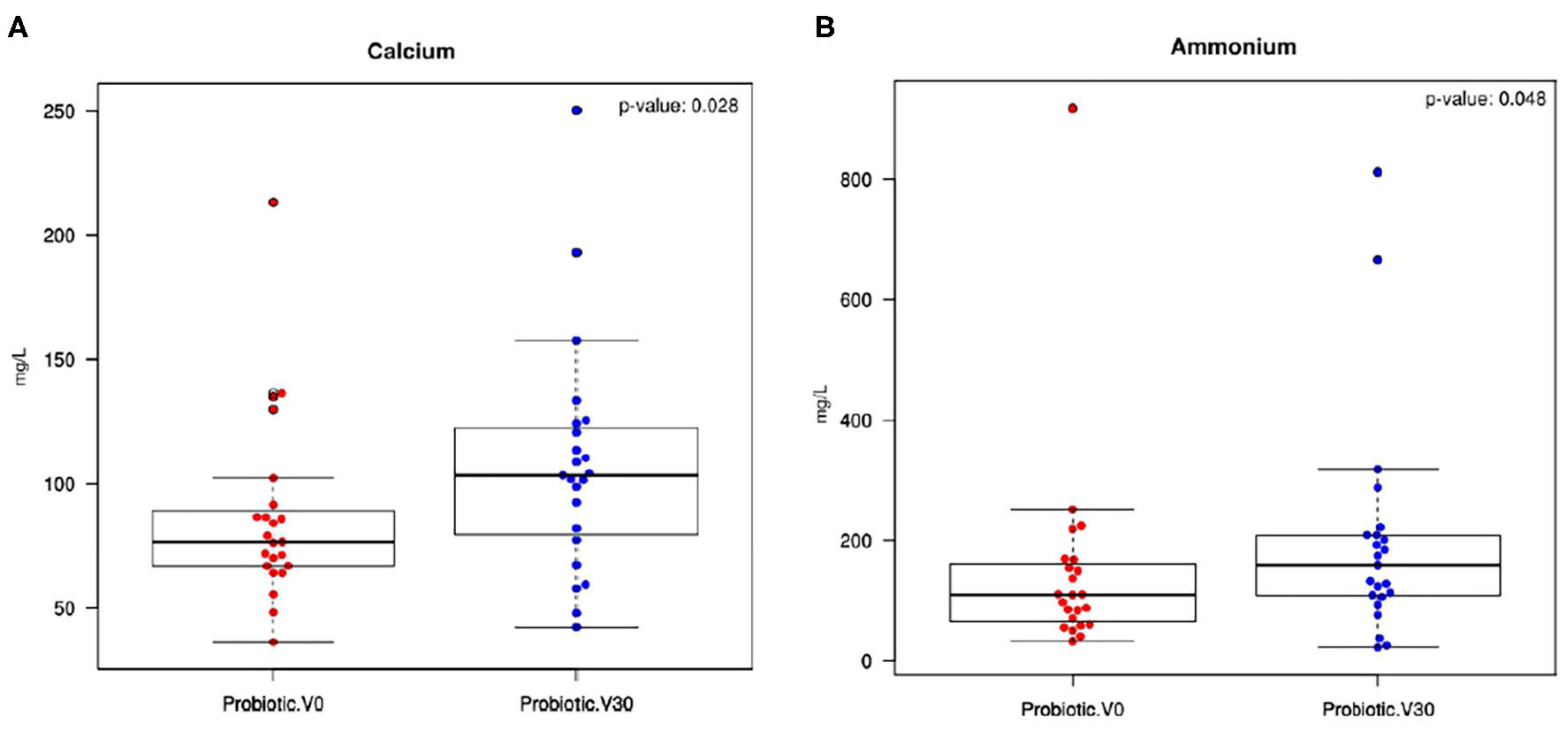
Calcium and ammonium salivary levels. Boxplots show unstimulated saliva concentration (mg/L) of **(A)** calcium and **(B)** ammonium in the probiotic group between baseline levels (Probiotic V0) and the end of treatment (probiotic V30). Wilcox test results for the comparison are also shown.

Interestingly, the electrolyte measures showed a significant increase in salivary calcium (*p* = 0.028) and ammonium concentrations (*p* = 0.048) between the beginning and the end of treatment in the probiotic group (Figures 4A,B, respectively). In fact, this increase in calcium and ammonium levels after the treatment period leads to a significant separation between visits in the probiotic group samples (probiotic V0 vs. probiotic V30), as shown by a CCA analysis based on saliva electrolytes composition (*p* = 0.022; Supplementary Figure 5).

### Effects on Acidogenicity and Buffer Capacity of Biofilm and Saliva

To investigate the possible impact of *S. dentisani* application on the acidogenicity of saliva and dental biofilm, the pH in saliva and plaque samples were measured at different time points in every visit. pH measurements in saliva samples were performed upon arrival to the clinic (“basal” samples), after plaque removal by brushing with water (pre-sugar samples) and 10 min after a sugar rinse with a 10% sucrose solution, as a measure of the buffering capacity in saliva (post-sugar samples). In the case of supragingival dental plaque, both pH and lactic acid measurements were measured *ex vivo* in basal conditions on the freshly collected dental plaque (“basal” measurements) and 10 min after incubation at 37°C on a 10% sucrose solution, as a measurement of plaque buffering capacity (“post-sugar” measurements).

**Figure 5:**
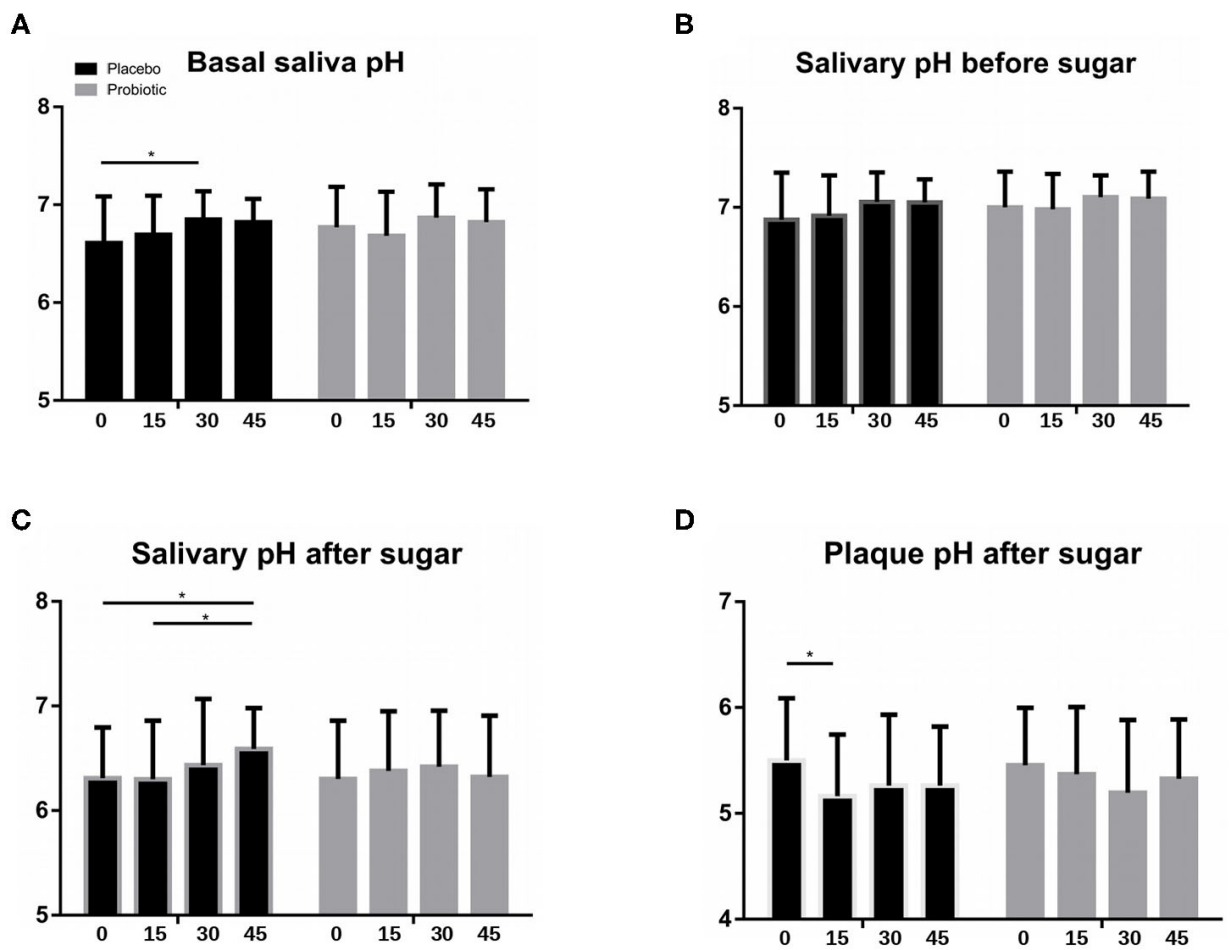
Salivary and plaque pH. Bars show mean values of **(A)** basal saliva pH (16–18 h. after last tooth-brushing), **(B)** salivary pH after plaque removal and before a sugar rinse, and **(C)** salivary pH 10 min after a sugar rinse, measured in unstimulated saliva samples from all patients in the probiotic (gray columns) and placebo (black) groups, at the four study visits (V0, V15, V30, and V45). **(D)** Mean values of plaque pH measured *ex vivo* 10 min after a 10% sugar pulse. Columns from left to right represent mean values (SD) at: V0, baseline visit; V15, 15 days during treatment; V30, end of treatment; and V45, 15 days after the end of treatment. *P < 0.05.

Figures 5A–C show the mean pH values of baseline, before-, and after-sugar samples, respectively, measured in saliva samples at the four study visits (V0, V15, V30, and V45). Average values for the three saliva samples at each visit were similar throughout the study, with no significant differences between groups. In the placebo group, a significant increase in the basal and after-sugar pH values were observed at V30 and V45, respectively (Figures 5A,C).

Plaque values of pH and lactic acid production did not vary between visits throughout the study in the probiotic group. In the placebo group however, there was a significant decrease in plaque pH values after 15 days of treatment (V15), both in basal (data not shown), and in after-sugar measurements (Figure 5D). Interestingly, a significant increase in plaque lactic acid production was observed on that visit (*p* = 0.04, Mann-Whitney test), both in basal and after-sugar samples (data not shown), suggesting that lactic acid production could be partly responsible for this pH drop at day 15.

### Saliva and Supragingival Dental Plaque Colonization of *S. dentisani*

**Figure 6:**
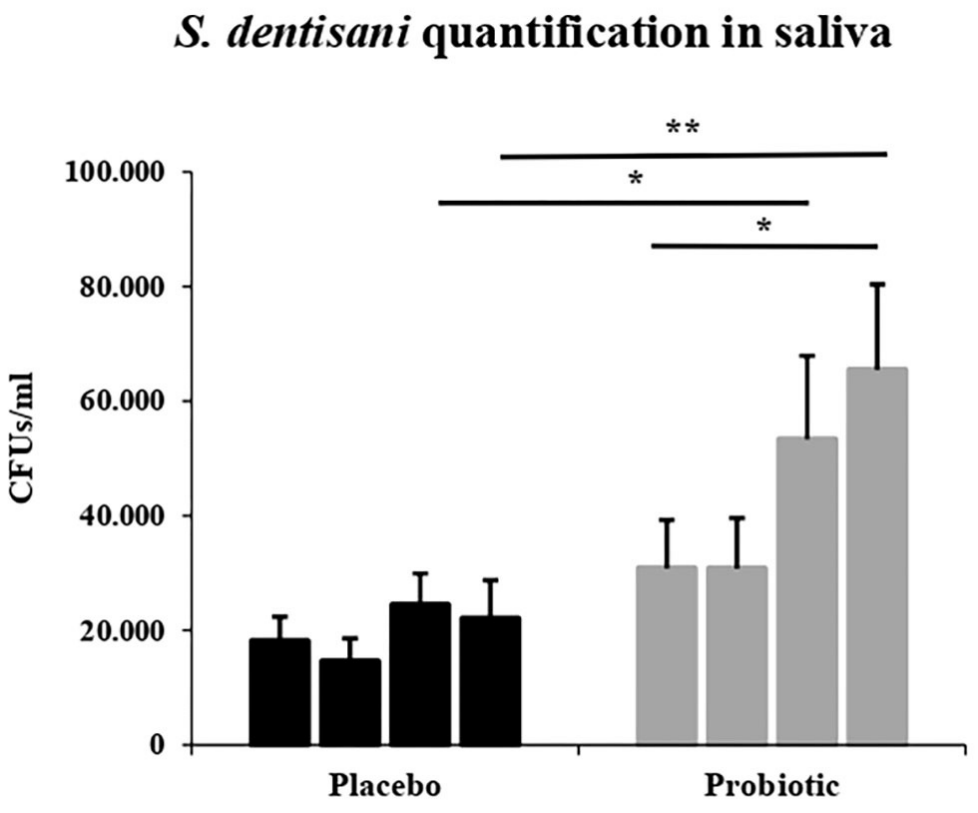
*Streptococcus dentisani* salivary levels. qPCR quantification of *S. dentisani* in saliva (number of bacterial cells/ml) throughout the study. Columns from left to right represent mean values (SEM) at: V0, baseline visit; V15, 15 days during treatment; V30, end of treatment; and V45, 15 days after the end of treatment in each study group (placebo in black and probiotic group in gray). *P < 0.05 and **P < 0.01.

The concentration of *S. dentisan*i in saliva samples was quantified by qPCR using species-specific primers, showing that mean values of cells/ml were significantly higher in the probiotic group compared to the placebo, both at the end of the treatment period (V30, *p* = 0.05) and at the end of the study (V45, *p* = 0.01). CFUs values at the end of the study in the probiotic group were also significantly higher (*p* = 0.02) compared to probiotic basal values (V0 vs.V45) (Figure 6).

Regarding the levels of the probiotic in dental plaque, a significant effect was found at baseline of the number of times the participants brushed their teeth on the levels of *S. dentisani*. Specifically, the number of tooth brushings per day significantly increased both the levels of *S. dentisani* normalized by ng of plaque (*p* = 0.03, Mann-Whitney test) and the percentage of *S. dentisani* relative to the total number of bacteria (*p* = 0.04), both estimated by qPCR (Supplementary Table 2). This suggests that *S. dentisani* plaque colonization is favored by plaque removal. In agreement with this, plaque levels increased throughout the clinical study not only in the probiotic group but also in the placebo (Supplementary Figure 6), likely as a consequence of the specific toothbrushing recommendations, material and training provided to the participants. However, as expected, the increase in *S. dentisani* plaque levels in those participants with colonization was significantly higher in the probiotic group compared to the placebo at the end of the treatment (*p* = 0.04). The proportion of participants with an increase in *S dentisani* levels in the probiotic group reached 58.3% by day 30 and 70.8% by day 45, indicating that plaque colonization can still be considerably improved.

## Discussion

The randomized, double-blind, placebo-controlled clinical study reported in the current manuscript has evaluated the effect of the probiotic *S. dentisani* strain 7746 buccoadhesive gel administration on several clinical and microbiological parameters, which are directly or indirectly related to oral health, specially tooth decay. Results are positive regarding safety and tolerability, and the analysis of data overall indicate that application of the probiotic improved parameters that are well-established markers of oral health. One of them is salivary flow, whose inverse relationship with the incidence of dental caries development is well-known (Rodríguez et al., 2015). In fact, a significant increase in dental caries risk has repeatedly been reported for patients taking drugs which reduce salivary flow or in patients with diseases where salivary glands are affected such as Sjögren's syndrome or head-neck cancer (Rodríguez et al., 2015). On the contrary, a higher salivary flow is associated with greater buffering capacity, salivary rinse (elimination and dilution of undesirable components), enhancement of the remineralization/demineralization ratio, increased antimicrobial action, and improved immune function (Dodds et al., 2015), leading to a less favorable environment for the development of cariogenic biofilms.

In the current work, although an improvement in salivary flow was observed in both treatment groups at the end of the study relative to baseline levels, the effect in the probiotic group was observed from the first visit, with a sialometry of up to one ml higher than the placebo group. The cause of that increase is difficult to determine as the smell and color of the placebo was indistinguishable from the probiotic. Our data on salivary ions suggest that the increase in salivary flow cannot be explained by changes in osmotic pressure, and future work should study if the probiotic has any effect on the complex physiological regulation of salivary function. Among the measured electrolytes, a significant increase in both calcium and ammonium salivary concentrations was detected between baseline and end-of-treatment values. Furthermore, the increase in these cations generated a significant change in the saliva composition, in terms of electrolyte concentration, at the end of the probiotic administration (Supplementary Figure 5). This increase in saliva calcium saturation induced by probiotic consumption could help in the delicate balance between remineralization and demineralization processes, on one hand by hampering the demineralization process during periods of low pH, and on the other by inducing dental remineralization when the pH returns to neutrality (Abou et al., 2016). This phenomenon can be beneficial for caries prevention, as illustrated by the work of Pandey et al. (2015), who observed significantly higher calcium salivary concentrations in caries-free compared to active caries children. This negative correlation between the incidence of dental caries and calcium salivary concentration is also reflected in different studies showing the beneficial effects of dairy probiotic products, as a consequence of the source of calcium provided by dairy food in general (Poureslami et al., 2013). To our knowledge, this is the first case where a direct association between an increase in salivary calcium levels and the administration of a non-dairy oral probiotic has been demonstrated, and future work should aim to understand the mechanism by which calcium concentrations are increased as a direct effect of a probiotic in the absence of dietary calcium supplementation.

The increase in ammonium after probiotic administration suggests that *S. dentisani* may provide higher buffering capacity to the oral biofilm. *S. dentisani* has been shown to efficiently metabolize arginine and produce extracellular ammonium by the so-called arginolytic pathway (López-López et al., 2017; Velsko et al., 2018). This ability of some oral bacteria to be able to produce ammonium from arginine or urea and release it extracellularly has been strongly related to caries-free individuals in both children and adults (Nascimento et al., 2009; Reyes et al., 2014). Thus, we propose that future laboratory and clinical studies should test the potential synergy between arginine supplementation and arginolytic probiotics to improve pH buffering capacity.

The clinical data show an improvement of plaque accumulation and gingival inflammation values associated to the probiotic treatment. This is of relevance for oral diseases, as both caries and periodontitis are biofilm-mediated diseases (Marsh, 2006). For example, higher plaque accumulation induces a larger pH drop after sugar intake and triggers gingival inflammation (Rosier et al., 2018). The gingival index improvement in the probiotic group is especially relevant because in this clinical study periodontal disease was an exclusion criterion, and therefore the starting gingival inflammation values were low, leaving little space for improvement. This, together with preliminary *in vitro* studies showing anti-inflammatory properties of *S. dentisani* and an inhibitory effect on the periodontal pathogen *Porphyromonas gingivalis* (Esteban-Fernández et al., 2018) suggests that future work should evaluate its effect on periodontal diseases through a study specifically focused on these patients.

The high-throughput sequencing of plaque samples provided a possible mechanism for the reduction in plaque accumulation and gingival inflammation. The probiotic treatment led to a significant decrease in the levels of *Fusobacterium nucleatum*. This is the bacterium with the largest degree of co-aggregation patterns in the oral cavity and is considered a “bridge” organism between early and late colonizers of this complex biofilm (Kolenbrander et al., 2010). In addition, the levels of *Corynebacterium matruchotii* at the end of the treatment period were significantly lower in the probiotic group than in the placebo group. Interestingly, recent fluorescent microscopy studies have shown that *C. matruchotii* is the keystone member of dental plaque architecture, forming long filaments from the tooth surface that serve as anchorage for many other oral inhabitants (Ferrer and Mira, 2016; Mark et al., 2016). Thus, both *F. nucleatum* and *C. matruchotii* are instrumental for oral biofilm formation and they therefore represent an ideal target for antimicrobial strategies in dental plaque control (Mira, 2018). Given that *S. dentisani* has shown *in vitro* inhibitory effects on both species (López-López et al., 2017; Llena et al., 2019), the reduction in plaque and gingival indexes in the current study suggests that *S. dentisani* could hinder the formation of mature plaque by the antagonistic action of bacteriocins (Mira, 2018). The reduction in *F. nucleatum* levels *in vivo* can also be relevant for systemic health, as this organism, in addition to being involved in plaque formation, periodontal disease and halitosis, has also been found to play a role in several other extraoral inflammatory processes such as sinusitis, endocarditis, septic arthritis, and abscesses of the brain between others, and recently has been shown to be involved in colorectal cancer (Han, 2015; Lee et al., 2019). Thus, we propose that future work aims to identify the specific bacteriocins which are responsible for the inhibition of this major human pathogen. On the other hand, a potentially negative effect of the *S. dentisani* strain 7746 bacteriocins effect is their inhibitory action against other *S. dentisani* strains (Conrads et al., 2018, 2019). Thus, although our data show an increase in *S. dentisani* levels after the administration of the probiotic, this could be so at the expense of a reduction in the indigenous population of this species.

The sequence data also allowed us to determine the microbiological impact derived from *S. dentisani* application. Results show that the probiotic treatment did not derive in an ecological catastrophe, with few significant changes in bacterial composition. One of those was of course an increase in *S. dentisani*, which was accompanied by a reduction in *Veillonella*. Given that the genus *Veillonella* uses organic acids as its only carbon source, it is frequently detected in dental caries lesions (Aas et al., 2008; Belda-Ferre et al., 2012) and its presence is associated with plaque acidogenicity. This could explain the increased levels of lactate in the placebo group at day 15. However, pH levels were not significantly different between the probiotic and placebo treatments, which could be due to the general improvement in this parameter throughout the study in both groups, probably as a consequence of efficient and constant toothbrushing when entering the trial. A limitation of the study is therefore the absence of a washout period before the treatment start, which may have eliminated the effect of toothbrushing in clinical parameters. Unexpectedly, the number of toothbrushings per day reported by participants at baseline correlated with *S. dentisani* levels at V0 (Supplementary Table 2). This, together with the increase in *S. dentisani* not only in the probiotic group but also in the placebo (Supplementary Figure 6), suggests that this species is favored by plaque removal, as it has been shown for other aerobic or facultatively anaerobic species in tongue biofilms (Tribble et al., 2019).

Other pathogens that had significantly lower levels after probiotic administration included *Scardovia wiggsiae*, an acidogenic organism strongly associated with early childhood caries (Eriksson et al., 2017), *Atopobium parvulum*, associated with caries and halitosis (Kazor et al., 2003), and other species associated with gingivitis or periodontitis like *Capnocytophaga leadbetteri* (Idate et al., 2018) or *Dialister invisus* (Downes et al., 2003). These changes, together with an increase of bacteria frequently associated with oral health such as *Gemella* sp. or *Kingella* sp. (Jiang et al., 2013; Chen C. et al., 2018; Chen W. P. et al., 2018) support that probiotic administration induced a shift in bacterial composition which was clearly associated with oral health. The species-level data derived from Illumina sequencing must nevertheless be taken with caution, as short fragments of the 16S rRNA gene may provide uncertain or wrong taxonomic assignments (Claesson et al., 2010). This is especially relevant for bacterial genera which are highly similar in the sequence of this gene, such as Streptococci. For example, *S. dentisani* is 100% identical in the V3–V4 regions of the 16S rRNA gene to *S. mitis* (Dzidic et al., 2018), and the levels of the probiotic must therefore be confirmed by qPCR, where specific primers provide a more accurate and realistic estimate of its levels (Conrads et al., 2018). However, this was not performed for other biologically relevant genera like *Fusobacterium* or *Capnocytophaga*, and the ASV analysis used in the current manuscript may therefore include incorrect species-level assignments.

Colonization is a crucial aspect in the evaluation of a probiotic product. *S. dentisani* has been found at high levels on supra and subgingival plaque, and at lower levels in saliva and tongue dorsum (Esteban-Fernández et al., 2019). In the current work, mean values of CFUs per ml of saliva were significantly higher in the probiotic group than those of placebo, both at the end of treatment and at the end of the study. In dental plaque, however, significant differences were only observed at the end of the application period, suggesting that colonization is partially lost after those 2 weeks, which is consistent with a previous pilot study where the probiotic was administered only for a week (Ferrer et al., 2020). Our data show that at the end of the treatment period, only 58% of participants in the probiotic group showed colonization, reaching 71% by the end of the study. Although it is possible that this poor colonization is influenced by host characteristics or lack of compliance, our data indicate that the dose, mode of application or galenic composition can probably be enhanced. Thus, the health-associated clinical and microbiological changes observed in the current study with levels of plaque colonization that can still be augmented suggest that the beneficial effects of *S. dentisani* could also be improved. It must nevertheless be kept in mind that 14 applications of a probiotic with mouth splints could be challenging for the patients and this should be improved in the future for simpler application protocols, but only if it does not affect the beneficial effects. In addition, it must also be considered that other oral streptococci have superior arginolytic activity (Velsko et al., 2018) and therefore the potential of different oral bacteria should be clinically tested as probiotics.

In conclusion, the topic application of *S. dentisani* appears to improve several clinical features associated with oral health, such as plaque accumulation, saliva quality, and salivary flow as well as an improvement of the disease-associated bacterial dysbiosis. Some of these improvements can be attributed to the antimicrobial activity of *S. dentisani*'s bacteriocins and to its pH-buffering capacity through the arginolytic pathway. However, the mechanistic cause underlying some of these changes (e.g., salivary flow) still need to be elucidated. We hope that the current manuscript stimulates future research to characterize the antimicrobial peptides arsenal of *S. dentisani* and to test the *in vivo* effect of other oral probiotics alone or in combination with prebiotics such as arginine that may enhance their effect.

## Data Availability Statement

The raw data supporting the conclusions of this article will be made available by the authors, without undue reservation.

## Ethics Statement

The studies involving human participants were reviewed and approved by Institutional Ethical Committee DGSP-CSISP. The patients/participants provided their written informed consent to participate in this study.

## Author Contributions

AM, CL, AL-L, and MF conceived and designed the study. MF, AL-L, TN, SP-V, AB-A, MD, and SG performed the experiments. MF and AL-L analyzed the data. AA and MF analyzed sequencing data. AM and CL provided reagents. MF, AL-L, and AM drafted the manuscript. All authors revised and approved the final manuscript.

## Conflict of Interest

AM is the inventor of a patent application protecting the use of *S. dentisani* as an oral probiotic. The remaining authors declare that the research was conducted in the absence of any commercial or financial relationships that could be construed as a potential conflict of interest.

## Supplementary Material

Supplementary Figure 1 Changes in clinical parameters. Data show means of increment values in **(A)** plaque index, **(B)** gingival index, and **(C)** salivary flow relative to baseline visit levels (V0). Columns from left to right represent increment mean values (SEM), both increase or decrease (Δ), from visit 0 (V0) to: V15, 15 days of treatment; V30, end of treatment; and V45, 15 days after the end of treatment.

Supplementary Figure 2 Oral microbiota composition of dental plaque. **(A)** Relative abundance of major bacterial genera identified in supragingival dental plaque samples before (V0) and after treatment (V30) as estimated by Illumina sequencing of the 16S rRNA gene. **(B)** Canonical Correspondence Analysis (CCA) of bacterial composition of plaque samples at the end of the treatment (V30) for each study participant. Each circle represents the structure in bacterial community composition of the probiotic group samples (in red) and those of the placebo (in blue).

Supplementary Figure 3 Shifts in bacterial levels of the most abundant organisms found in dental plaque samples. Boxplots represent the relative proportions of the most abundant genera **(A–C)** and species **(D,E)** between probiotic group samples at the end of treatment (probiotic V30) and the rest of samples (probiotic V0, placebo V0, and placebo V30), named in the figure as “others.” Wilcox test results for each comparison are also shown.

Supplementary Figure 4 Electrolytes concentration in saliva samples. Bars show the mean of **(A)** electrolytes sum (cations plus anions), **(B)** cations, and **(C)** anions separately, in unstimulated saliva samples, measured before (V0), and after treatment (V30). Columns from left to right represent mean values in mmol/L (SD) at: V0, baseline visit—and V30—end of treatment—in each treatment group (probiotic in black and placebo in gray).

Supplementary Figure 5 Salivary electrolytes composition within the probiotic group before and after the treatment period. Plot represents a Canonical Correspondence Analysis (CCA) of saliva samples in the probiotic group before (Probiotic V0) and after the 30-day treatment period (Probiotic V30) according to 11 electrolytes concentration (6 anions and 5 cations). Each circle represents the overall structure in saliva electrolytes composition of the probiotic group samples at baseline (in red) and those at the end of treatment (in blue). CCA *p*-value: 0.022.

Supplementary Figure 6 *S. dentisani* dental plaque colonization. Data represent *S. dentisani* cells, as estimated by qPCR quantifications in the colonized volunteers. Bars represent mean values (SEM) along the study period for the placebo (black) and probiotic (gray) groups. The number of colonized individuals in each time point is indicated. The asterisk indicates a significantly different colonization between the two groups (*p* < 0.05).

Supplementary Table 1 Mean proportions of dental plaque bacteria in placebo and probiotic groups at day 0 (basal visit) and day 30 (end of treatment visit) as determined by 16S rRNA gene Illumina sequencing.

Supplementary Table 2 Basal (indigenous) *Streptococcus dentisani* plaque levels for all participants in relation to toothbrushing frequency.
