# Supplementary figures and images for: Topic Application of the Probiotic Streptococcus dentisani Improves Clinical and Microbiological Parameters Associated With Oral Health
Source: Front Cell Infect Microbiol. 2020 Aug 31;10:465. doi: 10.3389/fcimb.2020.00465 (PMC7488176; doi:10.3389/fcimb.2020.00465)

Supplementary figure 1

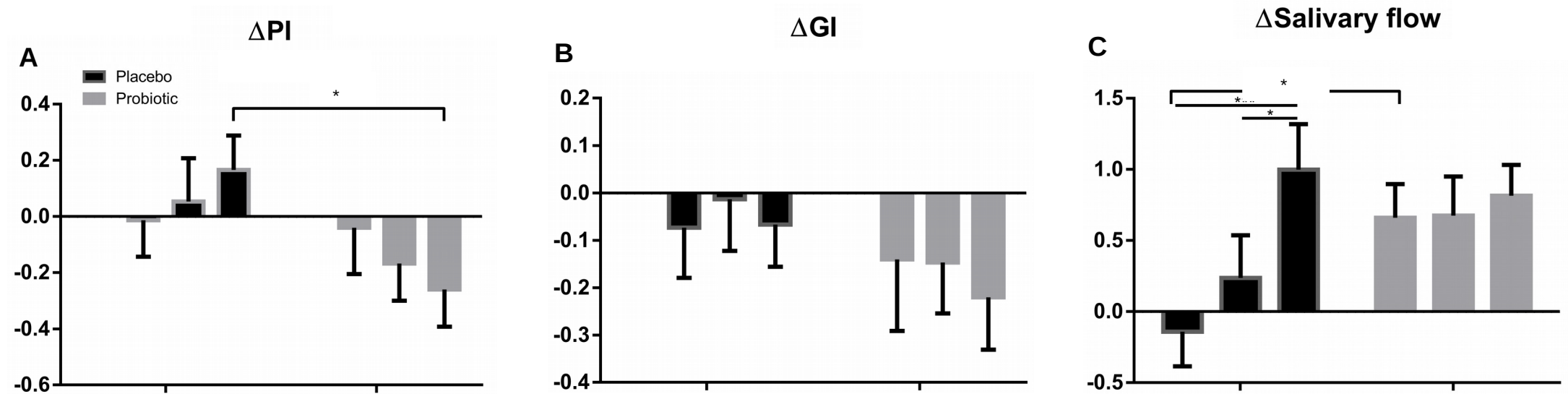

Supplement: Supplementary Figure 1 — Changes in clinical parameters. Data show means of increment values in (A) plaque index, (B) gingival index, and (C) salivary flow relative to baseline visit levels (V0). Columns from left to right represent increment mean values (SEM), both increase or decrease (Δ), from visit 0 (V0) to: V15, 15 days of treatment; V30, end of treatment; and V45, 15 days after the end of treatment. [file Data_Sheet_1.PDF]

Supplementary figure 2

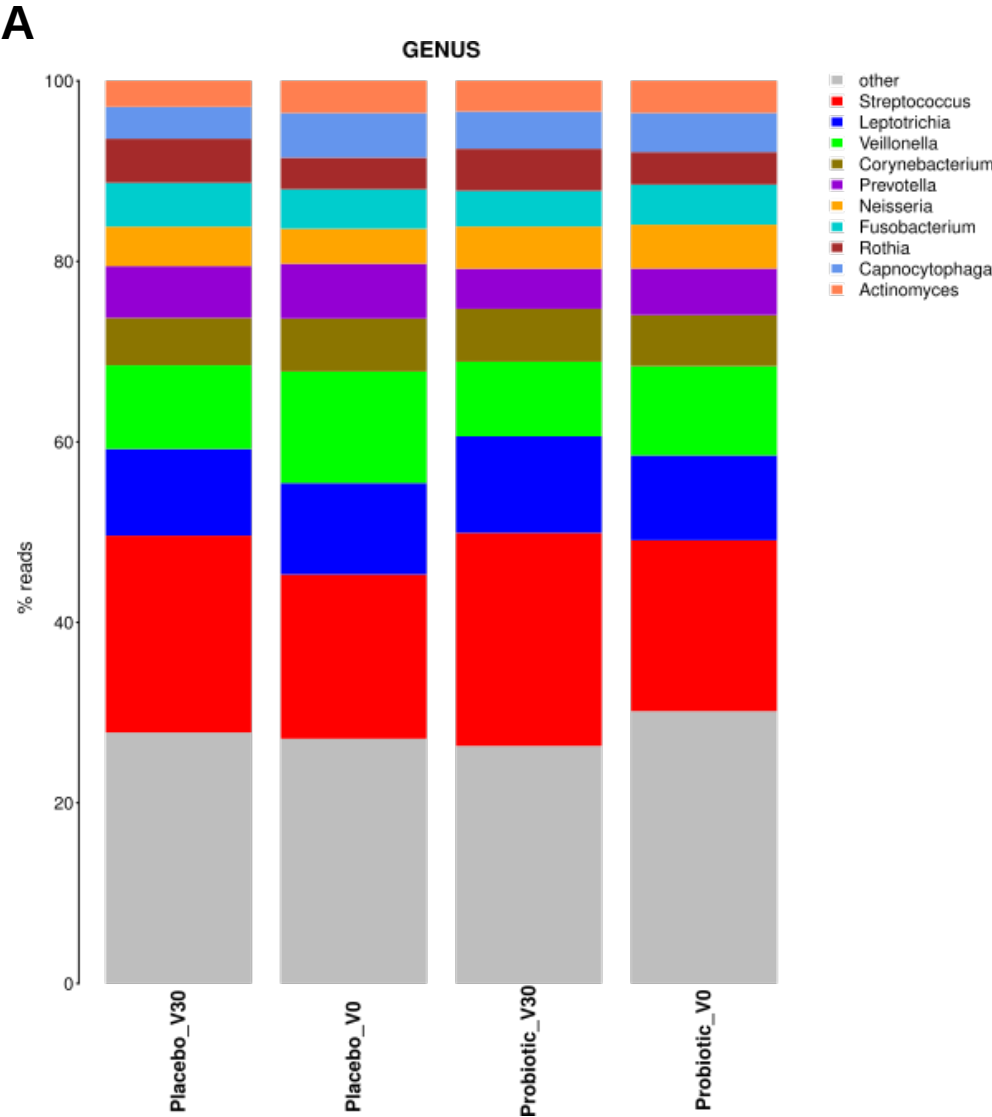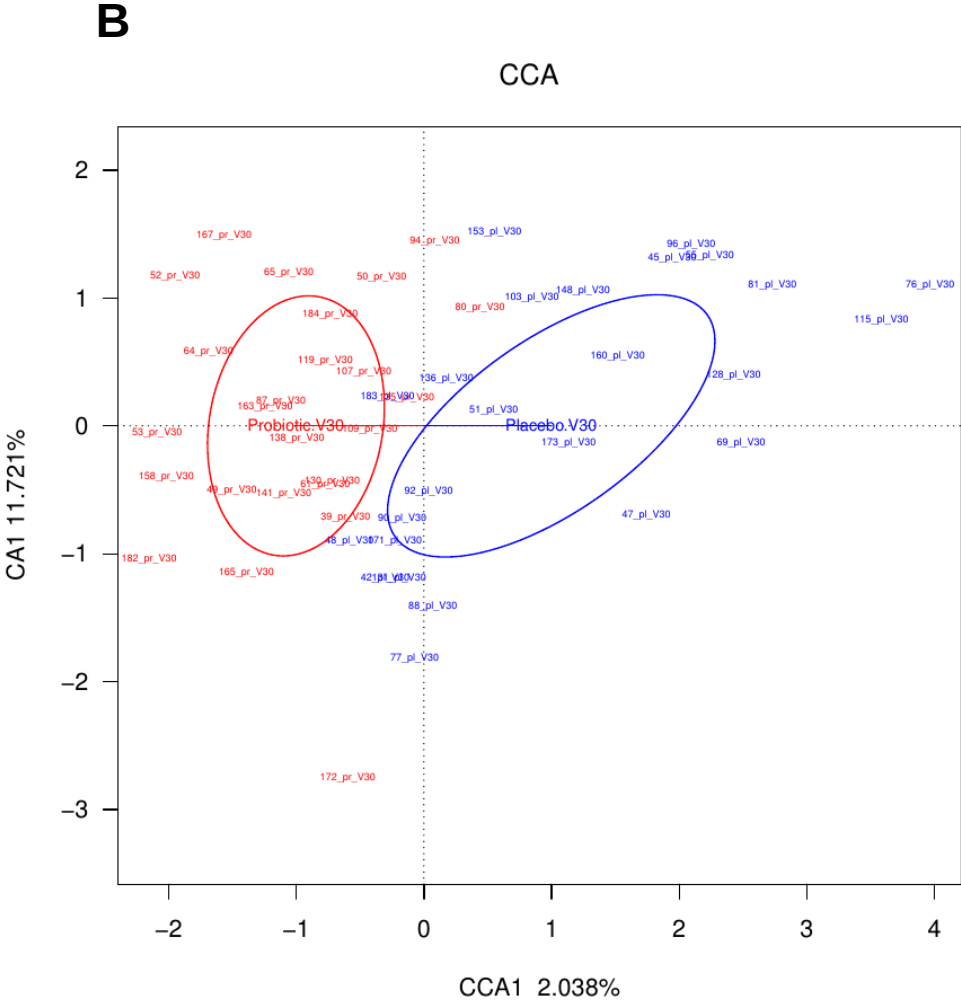

Supplement: Supplementary Figure 2 — Oral microbiota composition of dental plaque. (A) Relative abundance of major bacterial genera identified in supragingival dental plaque samples before (V0) and after treatment (V30) as estimated by Illumina sequencing of the 16S rRNA gene. (B) Canonical Correspondence Analysis (CCA) of bacterial composition of plaque samples at the end of the treatment (V30) for each study participant. Each circle represents the structure in bacterial community composition of the probiotic group samples (in red) and those of the placebo (in blue). [file Data_Sheet_2.PDF]

Supplementary figure 3

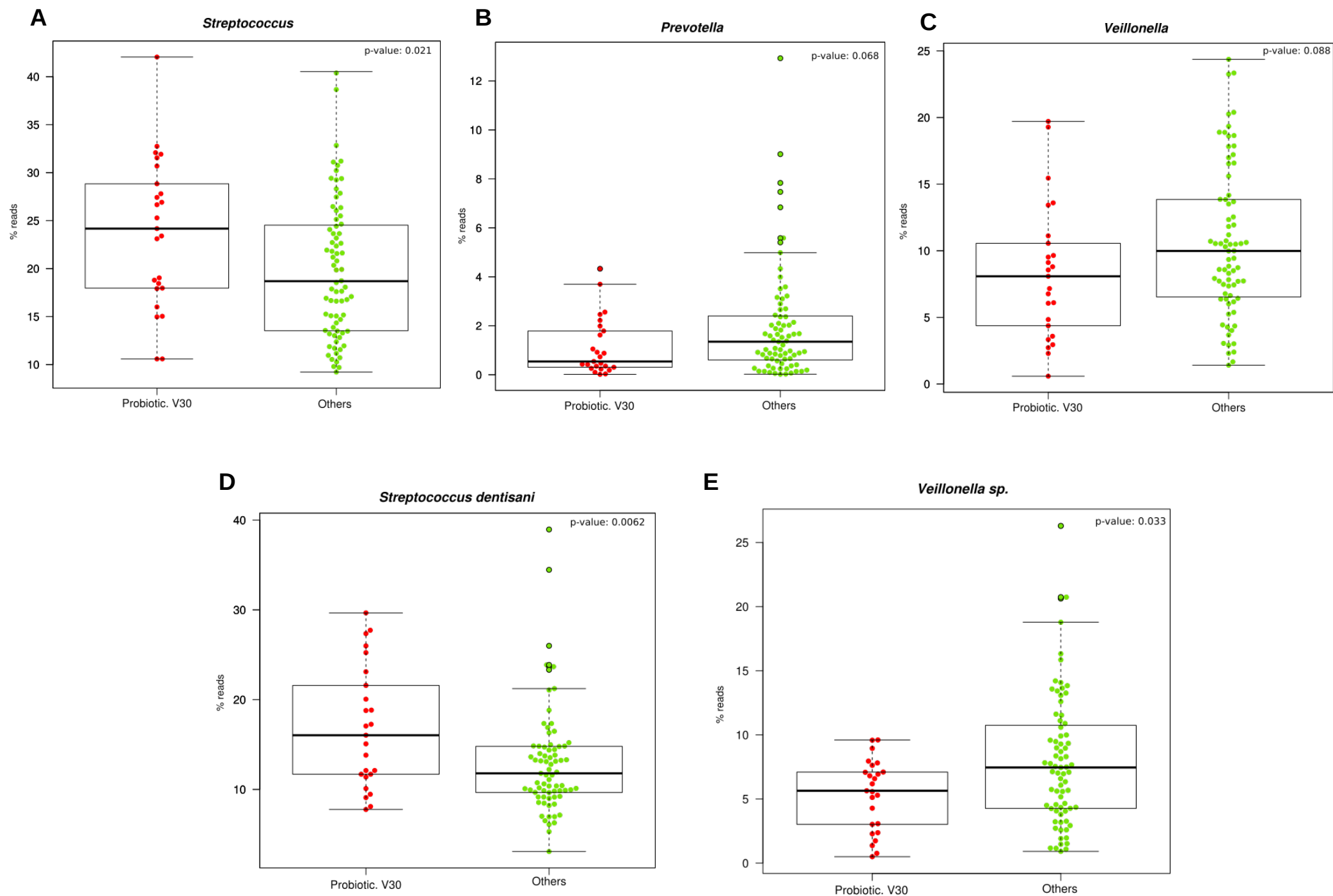

Supplement: Supplementary Figure 3 — Shifts in bacterial levels of the most abundant organisms found in dental plaque samples. Boxplots represent the relative proportions of the most abundant genera (A–C) and species (D,E) between probiotic group samples at the end of treatment (probiotic V30) and the rest of samples (probiotic V0, placebo V0, and placebo V30), named in the figure as “others.” Wilcox test results for each comparison are also shown. [file Data_Sheet_3.PDF]

Supplementary figure 4

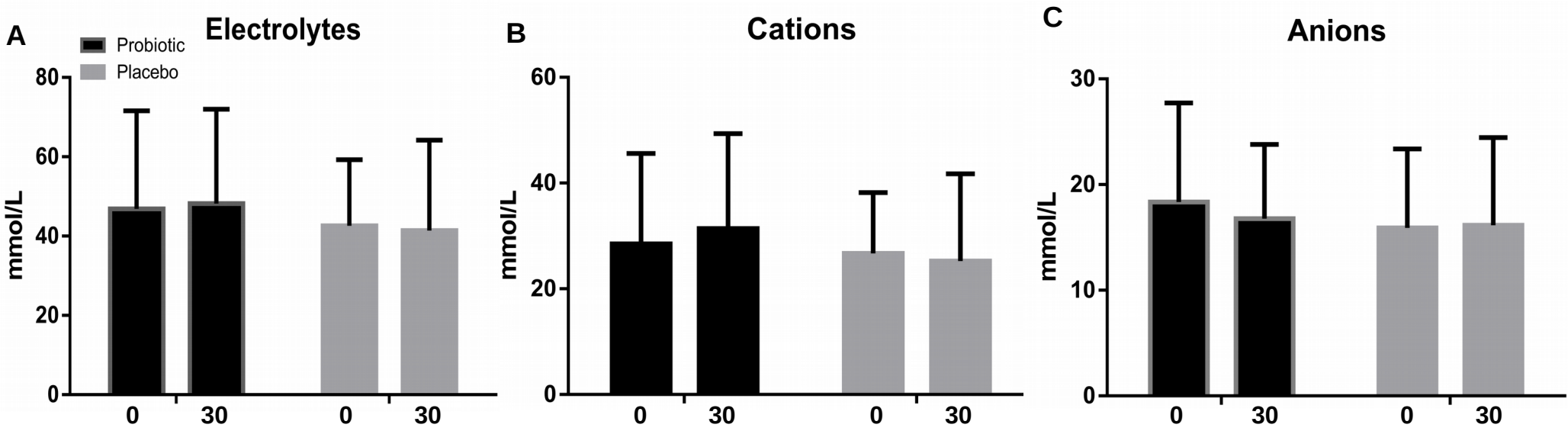

Supplement: Supplementary Figure 4 — Electrolytes concentration in saliva samples. Bars show the mean of (A) electrolytes sum (cations plus anions), (B) cations, and (C) anions separately, in unstimulated saliva samples, measured before (V0), and after treatment (V30). Columns from left to right represent mean values in mmol/L (SD) at: V0, baseline visit—and V30—end of treatment—in each treatment group (probiotic in black and placebo in gray). [file Data_Sheet_4.PDF]

### Supplementary figure 5

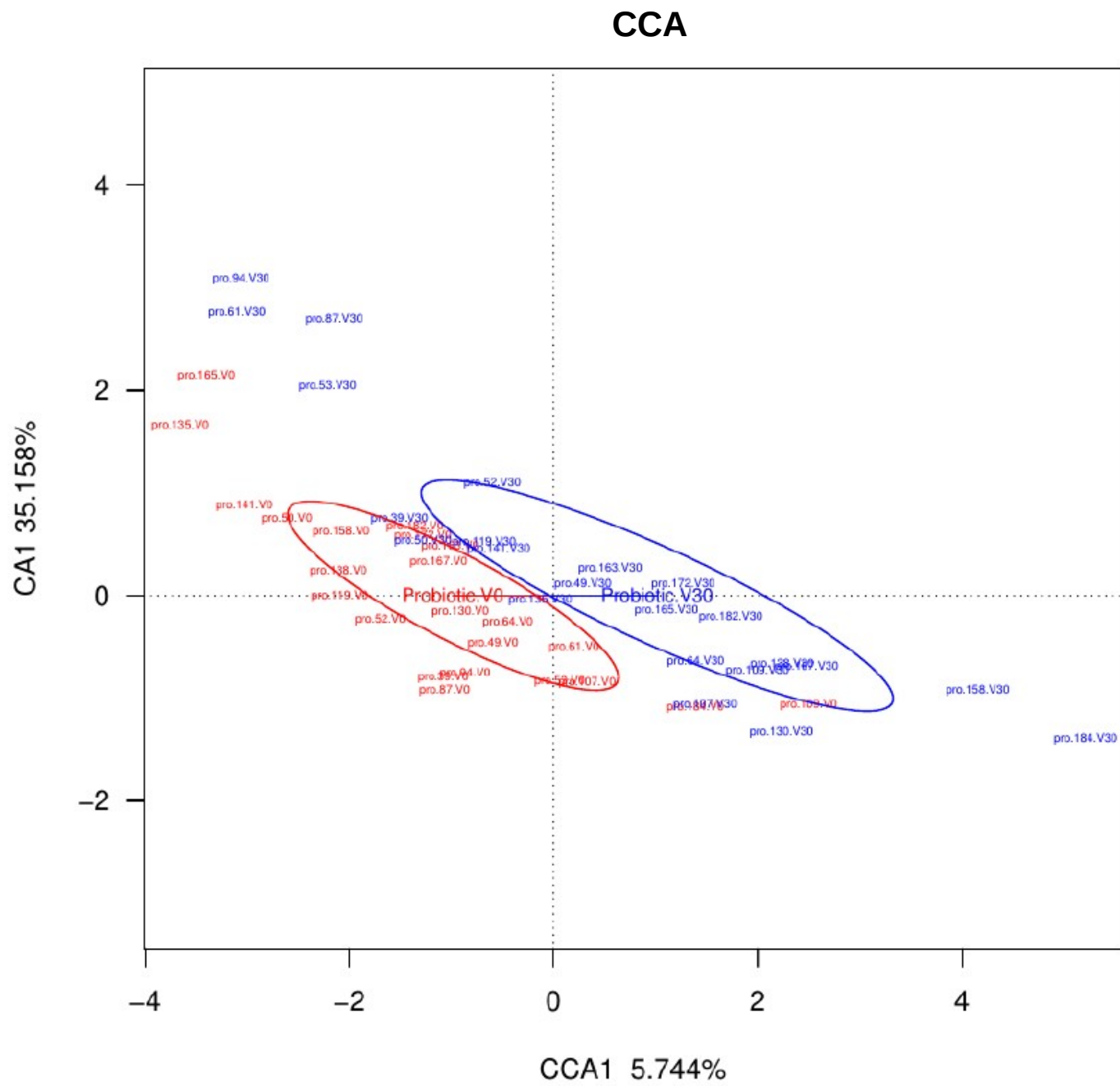

Supplement: Supplementary Figure 5 — Salivary electrolytes composition within the probiotic group before and after the treatment period. Plot represents a Canonical Correspondence Analysis (CCA) of saliva samples in the probiotic group before (Probiotic V0) and after the 30-day treatment period (Probiotic V30) according to 11 electrolytes concentration (6 anions and 5 cations). Each circle represents the overall structure in saliva electrolytes composition of the probiotic group samples at baseline (in red) and those at the end of treatment (in blue). CCA p-value: 0.022. [file Data_Sheet_5.PDF]

Supplementary figure 6

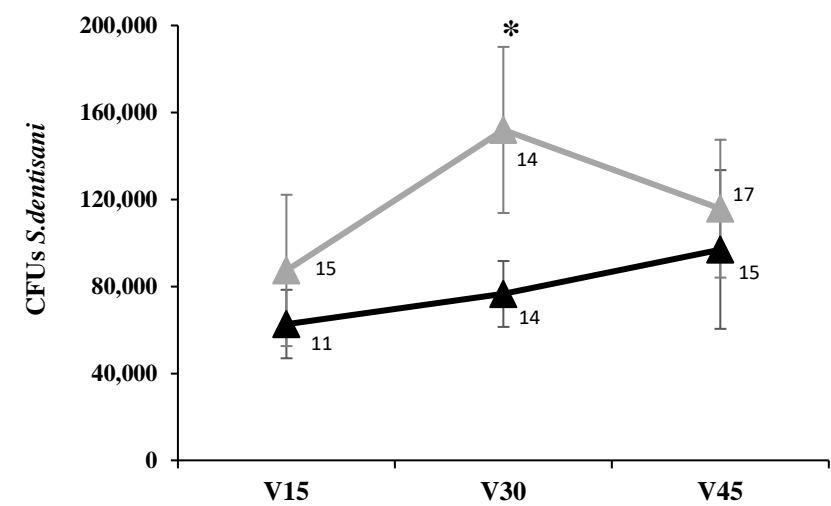

Supplement: Supplementary Figure 6 — S. dentisani dental plaque colonization. Data represent S. dentisani cells, as estimated by qPCR quantifications in the colonized volunteers. Bars represent mean values (SEM) along the study period for the placebo (black) and probiotic (gray) groups. The number of colonized individuals in each time point is indicated. The asterisk indicates a significantly different colonization between the two groups (p < 0.05). [file Data_Sheet_6.PDF]
