# Supplementary material for: Topic Application of the Probiotic Streptococcus dentisani Improves Clinical and Microbiological Parameters Associated With Oral Health
Source: Front Cell Infect Microbiol. 2020 Aug 31;10:465. doi: 10.3389/fcimb.2020.00465 (PMC7488176; doi:10.3389/fcimb.2020.00465)
Supplement: Supplementary Table 1 — Mean proportions of dental plaque bacteria in placebo and probiotic groups at day 0 (basal visit) and day 30 (end of treatment visit) as determined by 16S rRNA gene Illumina sequencing. [file Data_Sheet_7.PDF]

Supplementary Table 1. Mean proportions of dental plaque bacteria in placebo and probiotic groups at day 0 (basal visit) and day 30 (end of treatment visit) as determined by 16S rRNA gene Illumina sequencing.

DESCRIPTION *	Probiotic V0	Probiotic V30	Placebo V0	Placebo V30	ProbV0 vs ProbV30 p-values[1]	ProbV30 vs PlaceboV30 p-values[1]
p__Actinobacteria;c__Coriobacteriia;o__Coriobacteriales;f__Atopobiaceae;g__Atopobium	0.08	0.08	0.14	0.67	0.63	< 0.01
p__Actinobacteria;c__Actinobacteria;o__Bifidobacteriales;f__Bifidobacteriaceae;g__Scardovia	0.02	0.03	0.06	0.16	NA	0.01
p__Firmicutes;c__Negativicutes;o__Selenomonadales;f__Veillonellaceae;g__Megasphaera	0.04	0.04	0.08	0.18	NA	0.01
p__Patescibacteria;c__Saccharimonadia;o__Saccharimonadales;f__Saccharimonadaceae;g__Candidatus_Saccharimonas	0.08	0.05	0.09	0.15	0.58	0.01
p__Firmicutes;c__Negativicutes;o__Selenomonadales;f__Veillonellaceae;g__Dialister	0.23	0.21	0.30	0.58	0.48	0.01
p__Bacteroidetes;c__Bacteroidia;o__Flavobacteriales;f__Weeksellaceae;g__Bergeyella	0.74	0.69	0.60	0.43	0.94	0.04
p__Firmicutes;c__Clostridia;o__Clostridiales;f__Lachnospiraceae;g__Oribacterium	0.13	0.11	0.14	0.25	0.96	0.04
p__Firmicutes;c__Bacilli;o__Bacillales;f__Family_XI;g__Gemella	1.76	1.82	1.01	1.05	0.43	0.05
p__Firmicutes;c__Negativicutes;o__Selenomonadales;f__Veillonellaceae;g__Selenomonas_4	0.20	0.18	0.17	0.23	0.63	0.06
p__Actinobacteria;c__Coriobacteriia;o__Coriobacteriales;f__Atopobiaceae;g__Olsenella	0.05	0.06	0.10	0.15	0.61	0.07
p__Firmicutes;c__Negativicutes;o__Selenomonadales;f__Veillonellaceae;g__Selenomonas	0.38	0.37	0.40	0.63	0.08	0.09
p__Bacteroidetes;c__Bacteroidia;o__Bacteroidales;f__Porphyromonadaceae;g__Porphyromonas	2.98	2.46	2.05	1.82	0.43	0.10
p__Bacteroidetes;c__Bacteroidia;o__Bacteroidales;f__Prevotellaceae;g__Prevotella_7	1.66	1.11	2.14	2.13	0.08	0.10

p__Firmicutes;c__Bacilli;o__Lactobacillales;f__Carnobacteriaceae;g__Granulicatella	1.46	1.22	0.91	1.13	0.43	0.12
p__Firmicutes;c__Clostridia;o__Clostridiales;f__Lachnospiraceae;g__Stomatobaculum	0.20	0.18	0.17	0.21	0.31	0.14
p__Firmicutes;c__Negativicutes;o__Selenomonadales;f__Veillonellaceae;g__Anaeroglobus	0.11	0.06	0.12	0.27	NA	0.14
p__Firmicutes;c__Erysipelotrichia;o__Erysipelotrichales;f__Erysipelotrichaceae;g__Solobacterium	0.05	0.05	0.06	0.14	0.83	0.16
p__Proteobacteria;c__Gammaproteobacteria;o__Pasteurellales;f__Pasteurellaceae;g__Aggregatibacter	1.05	1.15	0.71	0.59	0.63	0.17
p__Spirochaetes;c__Spirochaetia;o__Spirochaetales;f__Spirochaetaceae;g__Treponema_2	0.23	0.22	0.29	0.42	0.72	0.22
p__Proteobacteria;c__Gammaproteobacteria;o__Betaproteobacteriales;f__Neisseriaceae;g__Neisseria	4.85	4.67	3.87	4.38	0.94	0.24
p__Bacteroidetes;c__Bacteroidia;o__Bacteroidales;f__Tannerellaceae;g__Tannerella	0.82	0.71	0.75	0.98	0.63	0.25
p__Proteobacteria;c__Gammaproteobacteria;o__Betaproteobacteriales;f__Neisseriaceae;g__Kingella	2.21	1.75	1.96	1.20	0.20	0.27
p__Firmicutes;c__Negativicutes;o__Selenomonadales;f__Veillonellaceae;g__Centipeda	0.02	0.04	0.06	0.06	NA	0.29
p__Bacteroidetes;c__Bacteroidia;o__Flavobacteriales;f__Flavobacteriaceae;g__Capnocytophaga	4.34	4.13	4.94	3.54	0.62	0.31
p__Firmicutes;c__Bacilli;o__Lactobacillales;f__Streptococcaceae;g__Streptococcus	18.93	23.61	18.20	21.80	0.00	0.33
p__Fusobacteria;c__Fusobacteriia;o__Fusobacteriales;f__Fusobacteriaceae;g__Fusobacterium	4.45	3.96	4.37	4.87	0.52	0.34
p__Bacteroidetes;c__Bacteroidia;o__Bacteroidales;f__Prevotellaceae;g__Prevotella	1.65	1.62	2.01	1.98	0.71	0.36

p__Firmicutes;c__Bacilli;o__Lactobacillales;f__Aerococcaceae;g__Abiotrophia	0.78	0.67	0.34	0.35	0.75	0.37
p__Epsilonbacteraeota;c__Campylobacteria;o__Campylobacterales;f__Campylobacteraceae;g__Campylobacter	1.39	1.40	1.75	1.35	0.54	0.40
p__Bacteroidetes;c__Bacteroidia;o__Bacteroidales;f__Prevotellaceae;g__Prevotella_6	0.30	0.17	0.14	0.09	0.49	0.41
p__Fusobacteria;c__Fusobacteriia;o__Fusobacteriales;f__Leptotrichiaceae;g__Leptotrichia	9.40	10.69	10.12	9.61	0.19	0.46
p__Proteobacteria;c__Gammaproteobacteria;o__Betaproteobacteriales;f__Burkholderiaceae;g__Ralstonia	0.01	0.01	0.00	0.01	0.04	0.48
p__Actinobacteria;c__Actinobacteria;o__Corynebacteriales;f__Corynebacteriaceae;g__Corynebacterium	5.67	5.86	5.88	5.26	0.81	0.53
p__Firmicutes;c__Clostridia;o__Clostridiales;f__Lachnospiraceae;g__Lachnoanaerobaculum	0.66	0.69	0.70	0.79	0.69	0.55
p__Proteobacteria;c__Gammaproteobacteria;o__Betaproteobacteriales;f__Neisseriaceae;g__Eikenella	0.17	0.21	0.21	0.17	0.31	0.56
p__Actinobacteria;c__Actinobacteria;o__Actinomycetales;f__Actinomycetaceae;g__Actinomyces	3.56	3.39	3.56	2.87	0.87	0.58
p__Firmicutes;c__Clostridia;o__Clostridiales;f__Ruminococcaceae;g__Ruminococcaceae_UCG-014	0.25	0.24	0.22	0.32	0.85	0.59
p__Bacteroidetes;c__Bacteroidia;o__Bacteroidales;f__Prevotellaceae;g__Prevotella_2	1.55	1.55	1.79	1.53	0.87	0.60
p__Firmicutes;c__Negativicutes;o__Selenomonadales;f__Veillonellaceae;g__Veillonella	9.88	8.26	12.35	9.26	0.13	0.69
p__Actinobacteria;c__Actinobacteria;o__Propionibacteriales;f__Propionibacteriaceae;g__Pseudopropionibacterium	0.05	0.09	0.04	0.07	0.07	0.71

p__Proteobacteria;c__Gammaproteobacteria;o__Pasteurellales;f__Pasteurellaceae;g__Haemophilus	3.66	2.27	4.02	2.76	0.03	0.71
p__Bacteroidetes;c__Bacteroidia;o__Bacteroidales;f__Prevotellaceae;g__Alloprevotella	0.62	0.43	0.64	0.54	0.31	0.71
p__Proteobacteria;c__Gammaproteobacteria;o__Cardiobacteriales;f__Cardiobacteriaceae;g__Cardiobacterium	0.82	0.74	1.03	0.77	0.29	0.74
p__Firmicutes;c__Negativicutes;o__Selenomonadales;f__Veillonellaceae;g__Selenomonas_3	2.08	1.93	2.15	2.20	0.56	0.74
p__Bacteroidetes;c__Bacteroidia;o__Bacteroidales;f__Paludibacteraceae;g__F0058	0.23	0.21	0.10	0.21	0.68	0.75
p__Firmicutes;c__Clostridia;o__Clostridiales;f__Peptococcaceae;g__Peptococcus	0.01	0.02	0.01	0.02	0.52	0.76
p__Proteobacteria;c__Gammaproteobacteria;o__Betaproteobacteriales;f__Burkholderiaceae;g__Lautropia	1.79	1.62	1.67	1.48	0.13	0.78
p__Proteobacteria;c__Gammaproteobacteria;o__Betaproteobacteriales;f__Burkholderiaceae;g__Comamonas	0.04	0.04	0.05	0.09	0.86	0.86
p__Firmicutes;c__Clostridia;o__Clostridiales;f__Family_XI;g__Parvimonas	0.11	0.19	0.13	0.23	0.20	0.88
p__Actinobacteria;c__Actinobacteria;o__Micrococcales;f__Micrococcaceae;g__Rothia	3.59	4.65	3.51	4.87	0.92	0.89
p__Synergistetes;c__Synergistia;o__Synergistales;f__Synergistaceae;g__Fretibacterium	0.05	0.02	0.04	0.05	0.86	0.91
p__Firmicutes;c__Clostridia;o__Clostridiales;f__Peptostreptococcaceae;g__Peptostreptococcus	0.03	0.04	0.02	0.07	0.22	0.95
p__Actinobacteria;c__Actinobacteria;o__Actinomycetales;f__Actinomycetaceae;g__F0332	0.37	0.33	0.40	0.32	0.60	0.98
p__Firmicutes;c__Clostridia;o__Clostridiales;f__Lachnospiraceae;g__Catonella	0.11	0.07	0.06	0.08	0.30	0.98

p__Firmicutes;c__Clostridia;o__Clostridiales;f__Lachnospiraceae;g__Johnsonella	0.30	0.25	0.21	0.27	0.94	0.98
p__Proteobacteria;c__Gammaproteobacteria;o__Pseudomonadales;f__Pseudomonadaceae;g__Pseudomonas	0.00	0.21	0.00	0.01	NA	NA
p__Proteobacteria;c__Gammaproteobacteria;o__Pseudomonadales;f__Moraxellaceae;g__Moraxella	0.10	0.09	0.01	0.01	NA	NA
p__Firmicutes;c__Clostridia;o__Clostridiales;f__Peptostreptococcaceae;g__Filifactor	0.02	0.02	0.02	0.05	NA	NA
p__Proteobacteria;c__Gammaproteobacteria;o__Pasteurellales;f__Pasteurellaceae;g__Actinobacillus	0.12	0.02	0.03	0.07	NA	NA
p__Firmicutes;c__Clostridia;o__Clostridiales;f__Lachnospiraceae;g__Shuttleworthia	0.00	0.00	0.00	0.09	NA	NA
p__Bacteroidetes;c__Bacteroidia;o__Bacteroidales;f__Bacteroidales_Incertae_Sedis;g__Phocaeicola	0.01	0.02	0.01	0.02	NA	NA
p__Proteobacteria;c__Gammaproteobacteria;o__Cardiobacteriales;f__Cardiobacteriaceae;g__Suttonella	0.01	0.00	0.01	0.02	NA	NA
p__Actinobacteria;c__Actinobacteria;o__Bifidobacteriales;f__Bifidobacteriaceae;g__Bifidobacterium	0.00	0.00	0.02	0.01	NA	NA
p__Firmicutes;c__Clostridia;o__Clostridiales;f__Peptostreptococcaceae;g__Peptoanaerobacter	0.02	0.01	0.01	0.01	NA	NA
p__Actinobacteria;c__Coriobacteriia;o__Coriobacteriales;f__Eggerthellaceae;g__Cryptobacterium	0.00	0.00	0.01	0.01	NA	NA
p__Proteobacteria;c__Gammaproteobacteria;o__Betaproteobacteriales;f__Rhodocyclaceae;g__Propionivibrio	0.01	0.01	0.00	0.01	NA	NA

p__Firmicutes;c__Clostridia;o__Clostridiales;f__Lachnospiraceae;g__Butyrivibrio_2	0.01	0.00	0.00	0.01	NA	NA
p__Firmicutes;c__Erysipelotrichia;o__Erysipelotrichales;f__Erysipelotrichaceae;g__Bulleidia	0.00	0.00	0.00	0.01	NA	NA
p__Bacteroidetes;c__Bacteroidia;o__Bacteroidales;f__Bacteroidaceae;g__Bacteroides	0.01	0.00	0.00	0.00	NA	NA
p__Firmicutes;c__Erysipelotrichia;o__Erysipelotrichales;f__Erysipelotrichaceae;g__Erysipelotrichaceae_UCG-006	0.00	0.00	0.00	0.01	NA	NA
p__Actinobacteria;c__Actinobacteria;o__Propionibacteriales;f__Propionibacteriaceae;g__Propionibacterium	0.00	0.00	0.00	0.01	NA	NA
p__Firmicutes;c__Clostridia;o__Clostridiales;f__Defluviitaleaceae;g__Defluviitaleaceae_UCG-011	0.01	0.00	0.00	0.00	NA	NA
p__Firmicutes;c__Clostridia;o__Clostridiales;f__Family_XIII;g__Family_XIII_UCG-001	0.00	0.00	0.00	0.01	NA	NA
p__Fusobacteria;c__Fusobacteriia;o__Fusobacteriales;f__Leptotrichiaceae;g__Oceanivirga	0.00	0.00	0.01	0.00	NA	NA
p__Firmicutes;c__Bacilli;o__Lactobacillales;f__Lactobacillaceae;g__Lactobacillus	0.03	0.00	0.01	0.00	NA	NA
p__Bacteroidetes;c__Bacteroidia;o__Bacteroidales;f__Rikenellaceae;g__Rikenellaceae_RC9_gut_group	0.00	0.00	0.01	0.01	NA	NA

* Taxonomic description ordered by significance of statistical test. p: Phylum; c: Class; o: Order; f: Family; g: Genus

[1] Wilcox text. NA: non-available
