# Supplementary material for: Topic Application of the Probiotic Streptococcus dentisani Improves Clinical and Microbiological Parameters Associated With Oral Health
Source: Front Cell Infect Microbiol. 2020 Aug 31;10:465. doi: 10.3389/fcimb.2020.00465 (PMC7488176; doi:10.3389/fcimb.2020.00465)
Supplement: Supplementary Table 2 — Basal (indigenous) Streptococcus dentisani plaque levels for all participants in relation to toothbrushing frequency. [file Data_Sheet_8.PDF]

**Supplementary Table 2**. Basal (indigenous) *Streptococcus dentisani* plaque levels for all participants in relation to toothbrushing frequency.

| | Toothbrushing Frequency[1] | Mean | Standard Deviation | p-value |
|---|---|---|---|---|
| *S. dentisani* levels[2] | 1 | $7.71x10^2$ | $1.07x10^3$ | 0.03 |
| | 2 | $1.43x10^3$ | $1.83x10^3$ | |
| | 3 | $1.42x10^3$ | $5.23x10^3$ | |
| *S. dentisani* %[3] | 1 | 0.29 | 0.35 | 0.04 |
| | 2 | 0.42 | 0.43 | |
| | 3 | 0.42 | 1.28 | |

[1] Number of tooth brushings per day as reported by participants at baseline

[2] Number of CFUs was calculated by qPCR and normalized by ng of DNA, as determined by fluorescence

[3] Percentage of the organism was calculated by qPCR with *S. dentisani* specific primers over the total number of bacteria as estimated by qPCR with universal primers
